# Supplementary material for: Seeding the meiotic DNA break machinery and initiating recombination on chromosome axes
Source: Nat Commun. 2024 Apr 5;15:2941. doi: 10.1038/s41467-024-47020-1 (PMC10997794; doi:10.1038/s41467-024-47020-1)
Supplement: Supplementary file 1 — Supplementary Information [file 41467_2024_47020_MOESM1_ESM.pdf]

SUPPLEMENTARY INFORMATION

Seeding the meiotic DNA break machinery and initiating recombination on chromosome axes

Author list:

Ihsan Dereli [1], Vladyslav Telychko [1], Frantzeskos Papanikos [1], Kavya Raveendran [1], Jiaqi Xu [2, 3], Michiel Boekhout [2], Marcello Stanzione [1], Benjamin Neuditschko [4], Naga Sailaja Imjeti [1], Elizaveta Selezneva [5], Hasibe Tuncay [6], Sevgican Demir [1], Teresa Giannattasio [7], Marc Gentzel [8], Anastasiia Bondarieva [1], Michelle Stevense [1], Marco Barchi [7], Arp Schnittger [6], John R. Weir [5], Franz Herzog [4], Scott Keeney [2, 3, 9], Attila Tóth [1*]

Affiliations

[1] Institute of Physiological Chemistry, Faculty of Medicine at the TU Dresden, Fiedlerstrasse 42 01307 Dresden, Germany

[2] Molecular Biology Program, Memorial Sloan Kettering Cancer Center, New York, NY 10065 USA

[3] Weill Cornell Graduate School of Medical Sciences, New York, NY 10065 USA

[4] Institute Krems Bioanalytics, IMC University of Applied Sciences, 3500 Krems, Austria

[5] Friedrich Miescher Laboratory of the Max Planck Society, Max-Planck-Ring 9, 72076 Tübingen, Germany.

[6] Department of Developmental Biology, University of Hamburg, 22609 Hamburg, Germany

[7] University of Rome "Tor Vergata", Section of Anatomy, Via Montpellier, 1, 00133, Rome, Italy.

[8] Core Facility Mass Spectrometry & Proteomics, Center for Molecular and Cellular Bioengineering (CMCB), Technische Universität Dresden, Dresden, Germany.

[9] Howard Hughes Medical Institute, Memorial Sloan Kettering Cancer Center, New York, NY 10065 USA

[*] Correspondence to attila.toth@mailbox.tu-dresden.de

SUPPLEMENTARY FIGURES AND TABLES

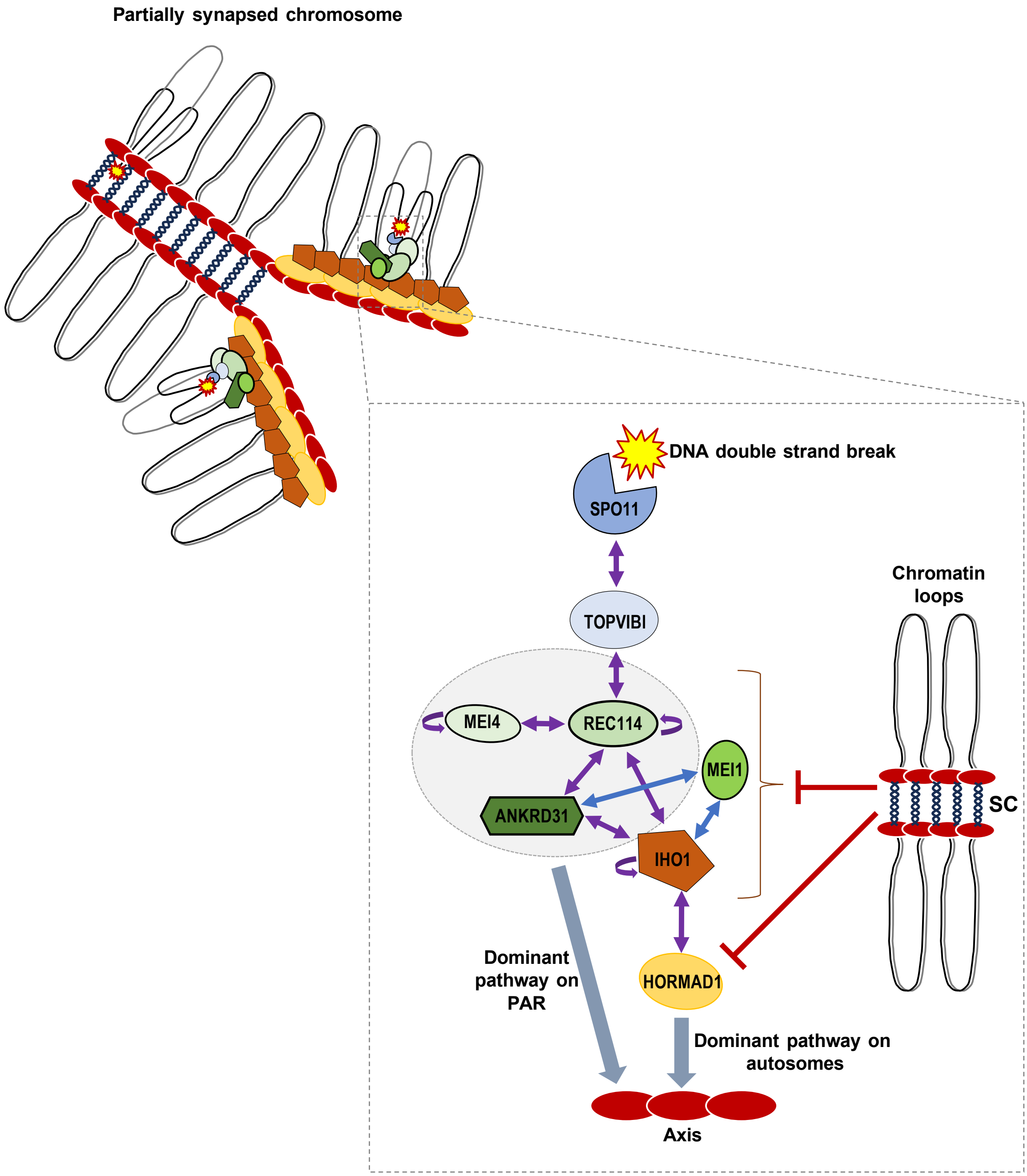

Supplementary Figure 1. Current model for the composition and the SC-induced downregulation of the axis-bound DSB machinery. Schematics show a partially synapsed chromosome where chromosome axes and the associated DSB machinery are represented. Enlarged inset shows physical interactions between DSB-machinery components (double headed arrows represent interactions that were previously published (purple) or are reported in this study (blue)), dominant pathways connecting the DSB-machinery to axes (grey-blue block arrows) and negative regulation of the DSB machinery (red blocking arrows) by the synaptonemal complex (SC) . Grey oval indicates the three DSB factors (MEI4, REC114 and ANKRD31) that were reported to have dominant roles in the assembly of the DSB-machinery on the pseudoautosomal regions (PAR) of sex chromosomes .

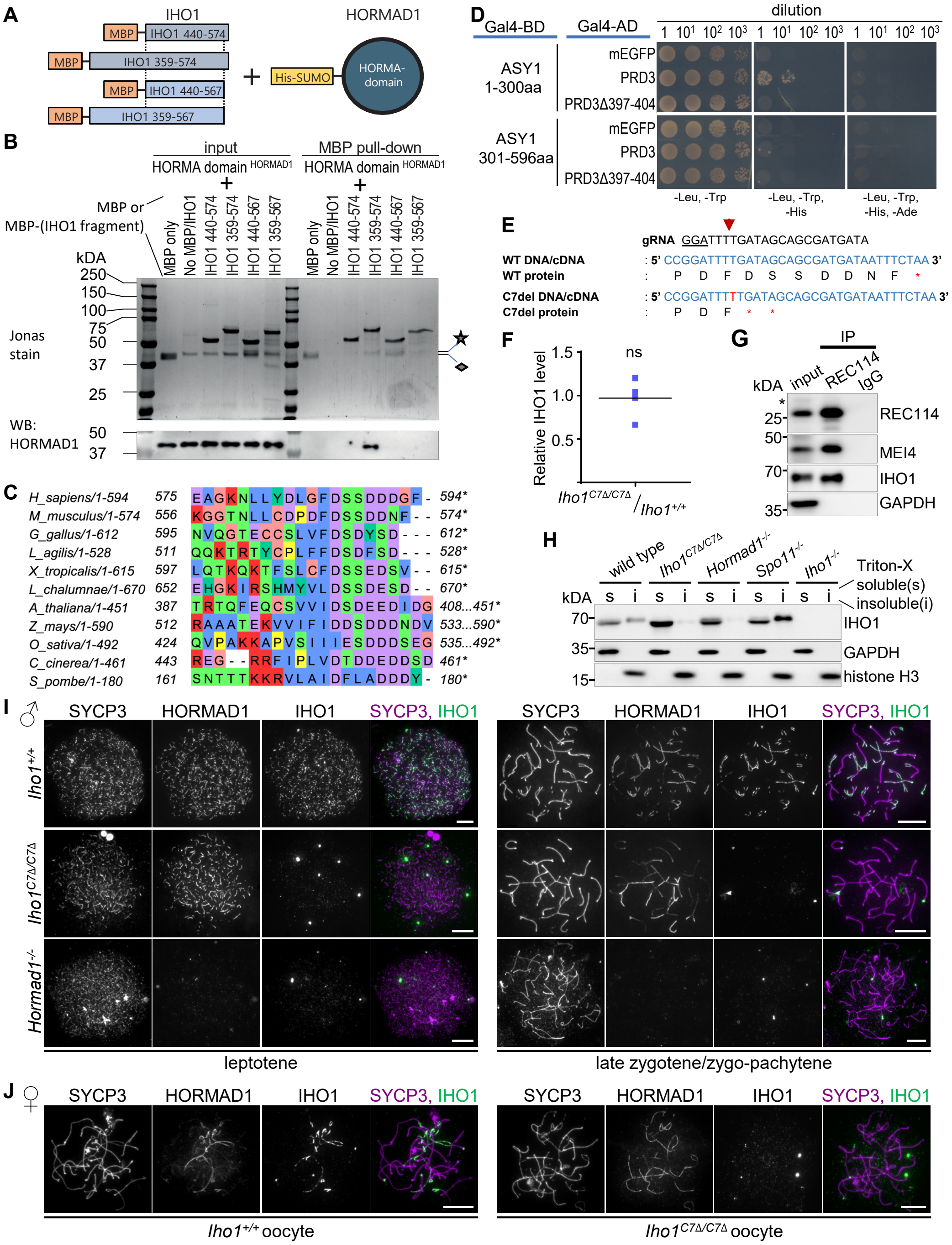

Supplementary Figure 2. A conserved motif in IHO1 C-terminus is required for axis localization of IHO1 in both sexes (relevant to Fig. 1) A Schematics of *in vitro* interaction assays between HORMA domain of HORMAD1 (1-240 amino acids) and fragments of IHO1 (the amino acid position of fragment boundaries are indicated). **B** Jonas stained gel picture (top) and immunoblot analysis (WB, bottom image) of *in vitro* MBP pull down assays testing interaction between purified His6-SUMO-tagged version of the HORMA domain of HORMAD1 (1-240 amino acids) and MBP-tagged versions of IHO1 fragments as depicted in **A**. Star and diamond mark the nearly indistinguishable positions of the protein bands of His6-SUMO-HORMAD1 HORMA domain (40.3kDa) and MBP, respectively. Note that the ~40kDa protein band that is detected in the MBP-IHO1 440-567 pull down by Jonas staining is probably a degradation product of MBP-IHO1 440-567 as it is not detected by the HORMAD1 immunoblot. **C** A window of a multiple sequence alignment of the last 100 amino acids of Mer2/IHO1-family proteins in diverse taxa, showing the previously described short similarity motif/SSM2 [10] and an overlapping/adjacent conserved acidic patch at its C-terminus. Amino acid positions of the beginning and the end of the window and the positions of the last amino acids (*) are shown in full length proteins. **D** Yeast two-hybrid interaction assays between *Arabidopsis thaliana* proteins. Interactions were tested between the N-terminal 300 amino acids of ASY1 (HORMAD1 orthologue) including the HORMA domain or a C-terminal ASY1 fragment (amino acid positions 301-596) containing a SWIRM domain and either wild-type PRD3 (IHO1 orthologue) or a PRD3 version that lacks the conserved IDSDEED motif (PRD3Δ397-404). Y2H assays between ASY1 fragments and mGFP served as negative controls. Cell suspensions of indicated optical densities (OD) were plated, and yeast cultures are shown after 6 days of growth on dropout plates. **E** Genomic DNA and corresponding protein sequence of IHO1-Cterminus in wild-type (WT) and *Iho1*$^{C7\Delta/C7\Delta}$ mutant line (C7del). Guide RNA sequence (gRNA) used for CRISPR/Cas9 editing is shown. PAM (underlined) and DNA cut site (red arrow) are marked. Red text color identifies mutated DNA and protein sequence, * marks STOP codons in the edited locus. **F** Quantification of total IHO1 protein levels in *Iho1*$^{C7\Delta/C7\Delta}$ mice. Immunoblot signals of total IHO1 protein from testis extracts of 13 dpp *Iho1*$^{C7\Delta/C7\Delta}$ mice were normalized to corresponding immunoblot signals of 13 dpp wild-type controls. Bar marks mean=0.97 of four experiments; two-tailed one-sample *t* test, ns=0.8055. **G** Immunoblots of immunoprecipitations (IP) from testis extracts of wild type mice at 13 dpp age. Immunoprecipitations with non-specific rabbit immunoglobulins (IgG) served as a negative control for IPs with rabbit anti-REC114 antibodies. Asterisk mark aspecific band that appears with variable intensity (compare to Fig. 1E) in REC114 immunoblots of IP-input testis extracts. Distinct proteins were detected on separate blots. **H** Immunoblot analysis of Triton X (0.3%) soluble (s) and insoluble (i) fractions of extracts of testes from 13 dpp mice of indicated genotypes. Panel shows IHO1, GAPDH (a marker of soluble fraction) and histone H3 (a marker of insoluble chromatin fraction). IHO1 and GAPDH were detected on the same blot, histone H3 was detected on a separate blot. **I-J** Immunofluorescence of IHO1, and markers of the chromosome axis (SYCP3) and unsynapsed axis (HORMAD1) in nuclear surface spread spermatocytes (**I**) of adult mice in indicated stages or late zygotene oocytes (**J**) of 16.5 days postcoitum (dpc) fetuses. Bars, 10 µm. Source data are provided as a Source Data file.

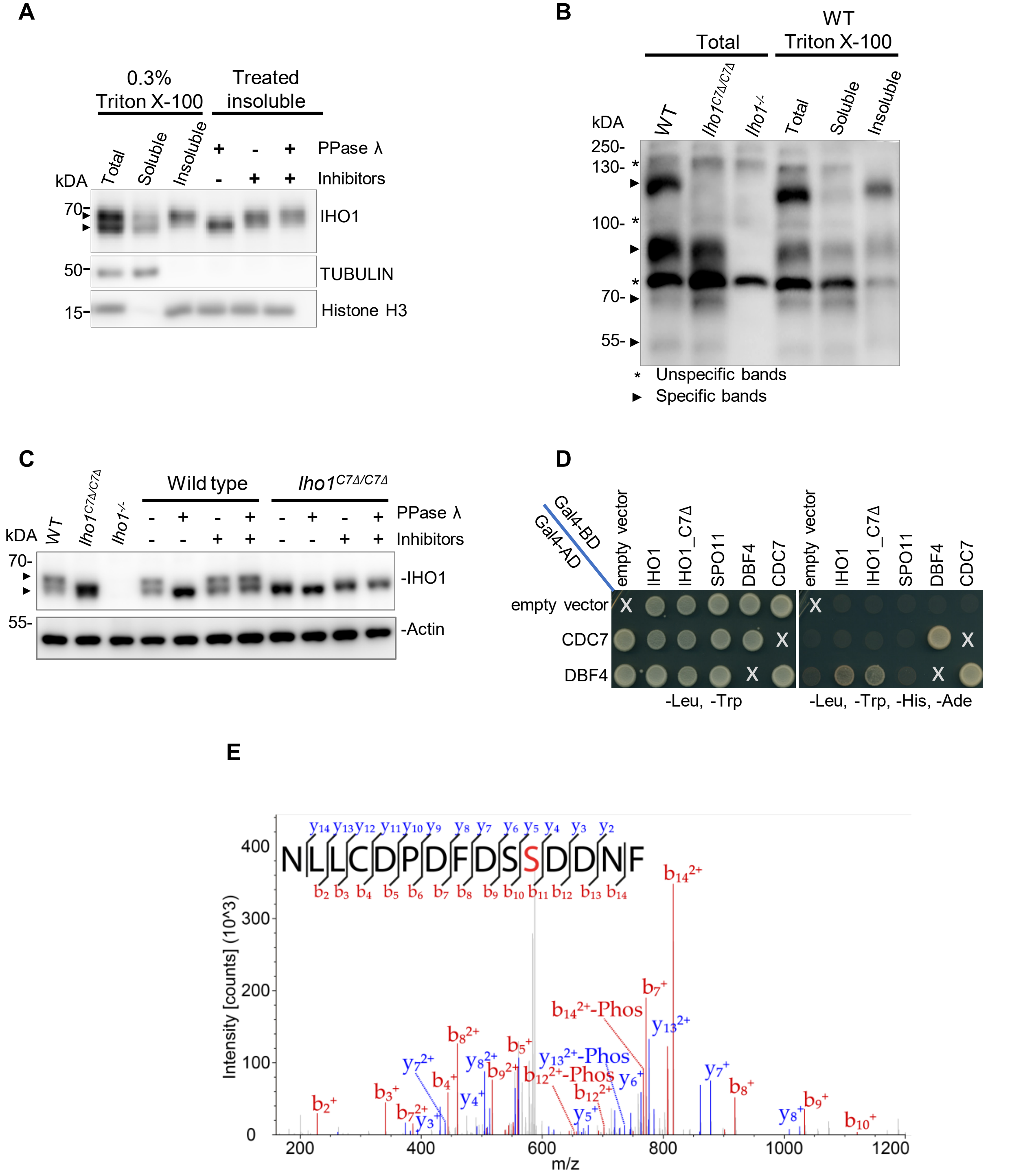

Supplementary Figure 3. Analyses of IHO1 phosphorylation, interactions between IHO1 and CDC7/DBF4 (relevant to Fig. 2)
A-C Immunoblots of standard (**A** and **C**) or phos-tag (**B**) SDS-PAGE of protein extracts from testes of 13 dpp mice. **A** Fractionation of testis extracts based on Triton X-100 solubility (left 3 lanes) and λ phosphatase treatment of Triton X 100-insoluble fraction (right 3 lanes) of wild-type testes. IHO1 and tubulin were detected on the same blot, histone H3 was detected on a separate blot. **B** Total testis extracts of mice with indicated genotypes (left 3 lanes) and fractionation of testis extracts of wild-type (WT, right 3 lanes) mice. Unspecific bands were marked by asterisk. Black triangles mark four distinct IHO1 specific bands that indicate an unphosphorylated form plus at least three phosphorylated forms for wild-type IHO1 and an unphosphorylated form plus at least two residual phosphorylated forms for IHO1_C7Δ. **C** Phosphatase treatment of total testis extracts from wild-type and *Iho1*$^{C7\Delta/C7\Delta}$ mice. IHO1 and actin were detected on the same blot. **A** and **C**, black triangles mark prominent slow and fast migrating forms of IHO1. **D** Yeast two-hybrid interaction assays between indicated proteins. Yeast cultures are shown after 3 days of growth on dropout plates. For negative control, proteins of interest were tested in transformations where either the Gal4-binding domain (Gal4-BD) or the Gal4-activation domain (Gal4-AD) vectors were empty. X marks bait-prey combinations that were omitted from Y2H due to lack of relevance. **E** MS/MS spectrum of NLLCDPDFDS(pS)DNF-NH_2 peptide. Identified b and y ions are annotated in red and blue, respectively. Fragments with neutral loss of H_3PO_4 are indicated as “-Phos”. 9+/-1.4% of the peptide was phosphorylated in n=3 measurements. Source data are provided as a Source Data file.

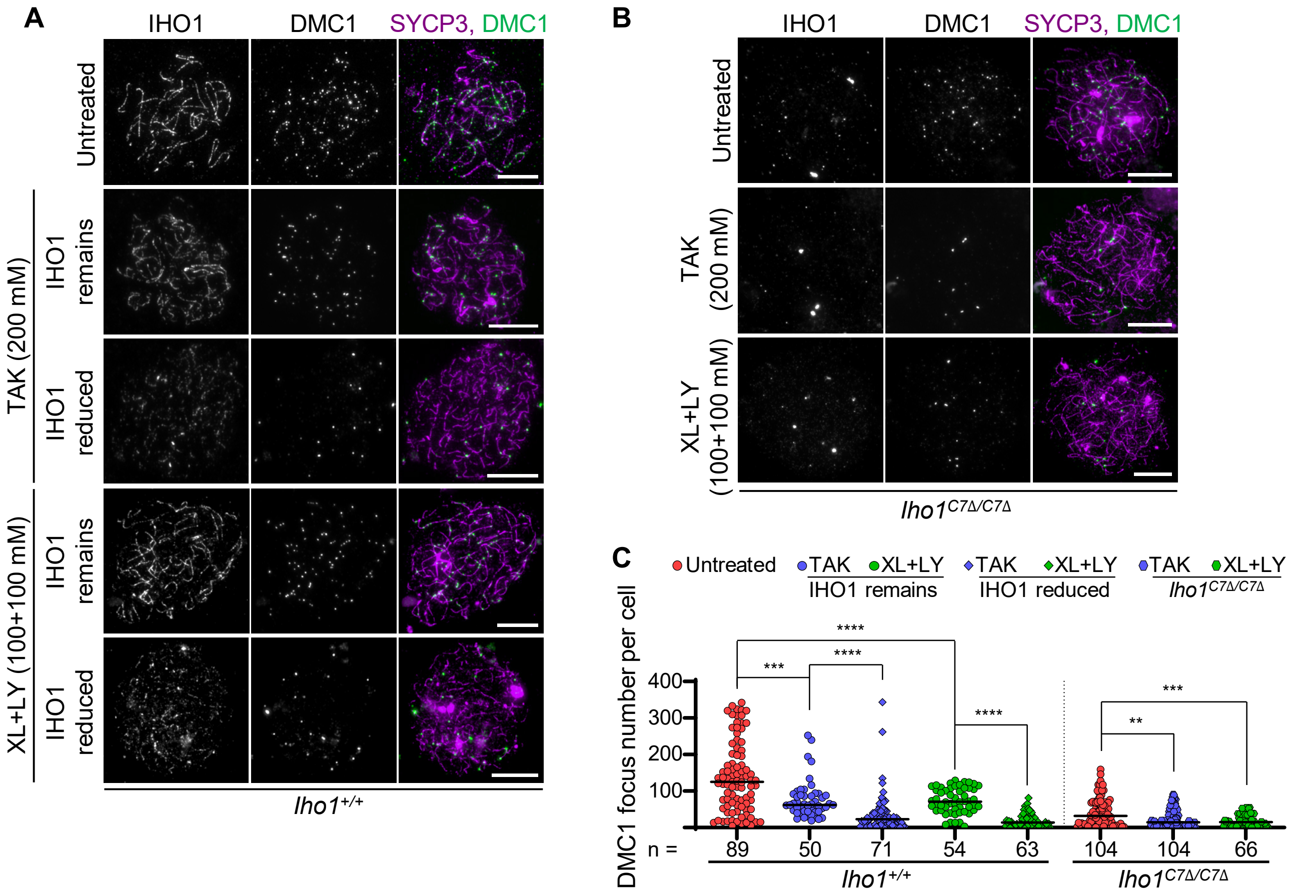

Supplementary Figure 4. Phenotypes of CDC7 inhibition in meiosis (relevant to Fig. 2)
Analysis of spermatocytes from testes of 8 dpp mice after 48 hours *in vitro* culture with or without CDC7 inhibitors 200mM TAK-931 (TAK) or a mix of 100mM XL413 and 100mM LY3143921 (XL+LY). **A-B** Immunofluorescence of IHO1, markers of the chromosome axis (SYCP3) and unrepaired DSBs (DMC1) in nuclear surface spread spermatocytes of *Iho1*$^{+/+}$ (**A**) or *Iho1*$^{C7\Delta/C7\Delta}$ (**B**) mice. **A** Spermatocytes are shown with normal or reduced levels of axial IHO1 in CDC7 inhibitor treated *Iho1*$^{+/+}$ samples. **C** Quantification of DMC1 foci, datapoints represent cells from 2 pooled experiments/mice. DMC1 quantifications are shown separately for *Iho1*$^{+/+}$ cell populations where IHO1 remained on axes and cell populations where IHO1 was depleted from axes following treatment with CDC7 inhibitors. Bars, medians. Two-tailed Mann-Whitney U test, s**=P<0.01, s***=P<0.001, s****=P<0.0001. Exact P values: for wild type spermatocytes, untreated vs. TAK treated IHO1 remains P=0.0001844, TAK treated IHO1 remains vs. TAK treated IHO1 reduced P=1.16e-9, untreated vs. XL+LY treated IHO1 remains P=4.78e-5, XL+LY treated IHO1 remains vs. XL+LY treated IHO1 reduced P=2.32e-14, for *Iho1*$^{C7\Delta/C7\Delta}$, untreated vs. TAK treated P=0.002149, untreated vs. XL+LY treated P=0.0002726. Source data are provided as a Source Data file.

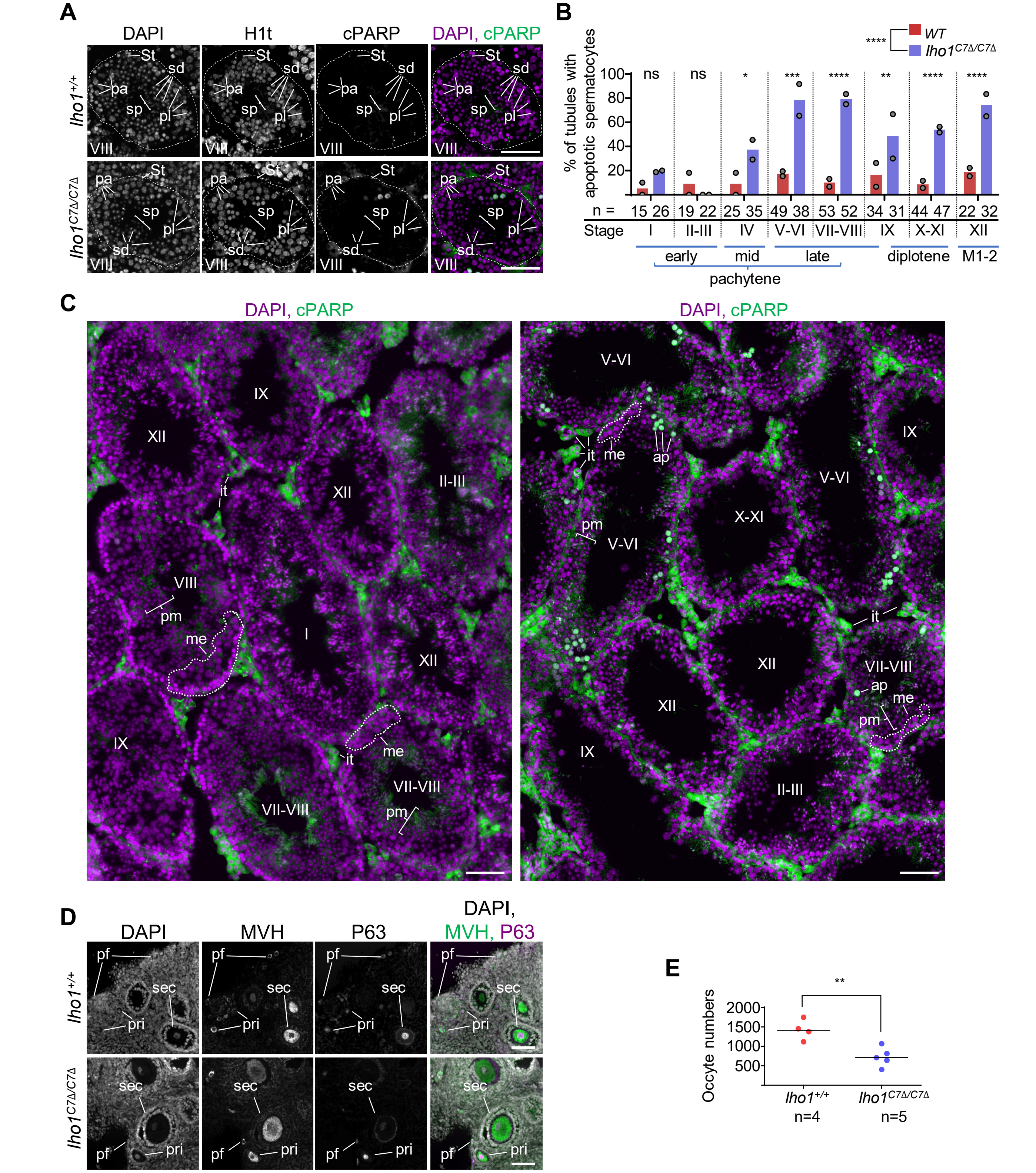

Supplementary Figure 5. *Iho1$^{C7\Delta/C7\Delta}$* mice show increased loss of meiocytes (relevant to Fig. 3)

A, C Sections of testes from adult *Iho1$^{+/+}$* and *Iho1$^{C7\Delta/C7\Delta}$* mice. DNA was detected by DAPI. Histone H1t (staging marker of seminiferous tubules, not shown in **C**) and cleaved PARP (cPARP, marker of apoptosis) were detected by immunostaining. Epithelial cycle stages of the seminiferous tubules are indicated by roman numbers. **A** Preleptotene (pl), pachytene (pa), round spermatid (sd), sperm (sp) and Sertoli cells (St) and outlines of tubules (white dashed line) are marked in a single seminiferous tubule in each genotype. **C** Multiple seminiferous tubules are shown for overview. Groups of meiotic cells (me with dashed outline), postmeiotic cells (pm), apoptotic meiotic cells/nuclei (ap), and inter-tubular somatic cells are pointed out in and next to two seminiferous tubules. Note that green signal in the cytoplasm of inter-tubular cells and the lumen of seminiferous tubules reflects nonspecific staining. **B** Quantification of seminiferous tubules that contain cPARP-positive apoptotic spermatocytes in the pachytene/diplotene/mitotically dividing cell layers. Block bars show the averages from two experiments, gray circles represent single experiments. One mouse represented each genotype in each experiment. n = number of tubules counted in two independent experiments. Analysis of deviance using the likelihood-ratio test based on the chi-squared distribution, ns=P>0.05, s*=P<0.05, s**=P<0.01, s***=P<0.001, s****=P<0.0001. Exact P values: Stage I P=0.2483, Stage II-III P=1, Stage IV P=0.01242, Stage V-VI P=1.14e-7, Stage VII-VIII P=7.85e-15, Stage IX P=0.006284, Stage X P=2.54e-6, Stage XI-XII P=6.42e-5, wild type vs. *Iho1$^{C7\Delta/C7\Delta}$* when all stages pooled P<2.2e-16. Most advanced meiotic cell-division cycle stage is indicated in the epithelial cycle stages. **D** Cryo sections of ovaries from young adult mice (6 weeks). Two oocyte markers, MVH (cytoplasmic) and p63 (nuclear) were immunostained, DNA was labeled by DAPI. Primordial (pf), primary (pri) and secondary (sec) follicles are indicated. **A, C, D** Bars, 100μm (**A** and **C**) and 20 μm (**D**). **E** Quantification of oocyte numbers in ovary sections from 6-weeks-old females of indicated genotypes. Sums of oocyte numbers from every 6th sections of both ovaries of each mouse are shown. *n* = numbers of analyzed animals; two-tailed Welch t-test, s**=P=0.005524. Bars mark mean numbers of oocytes (wild-type, 1424.25, *Iho1$^{C7\Delta/C7\Delta}$*, 728). Source data are provided as a Source Data file.

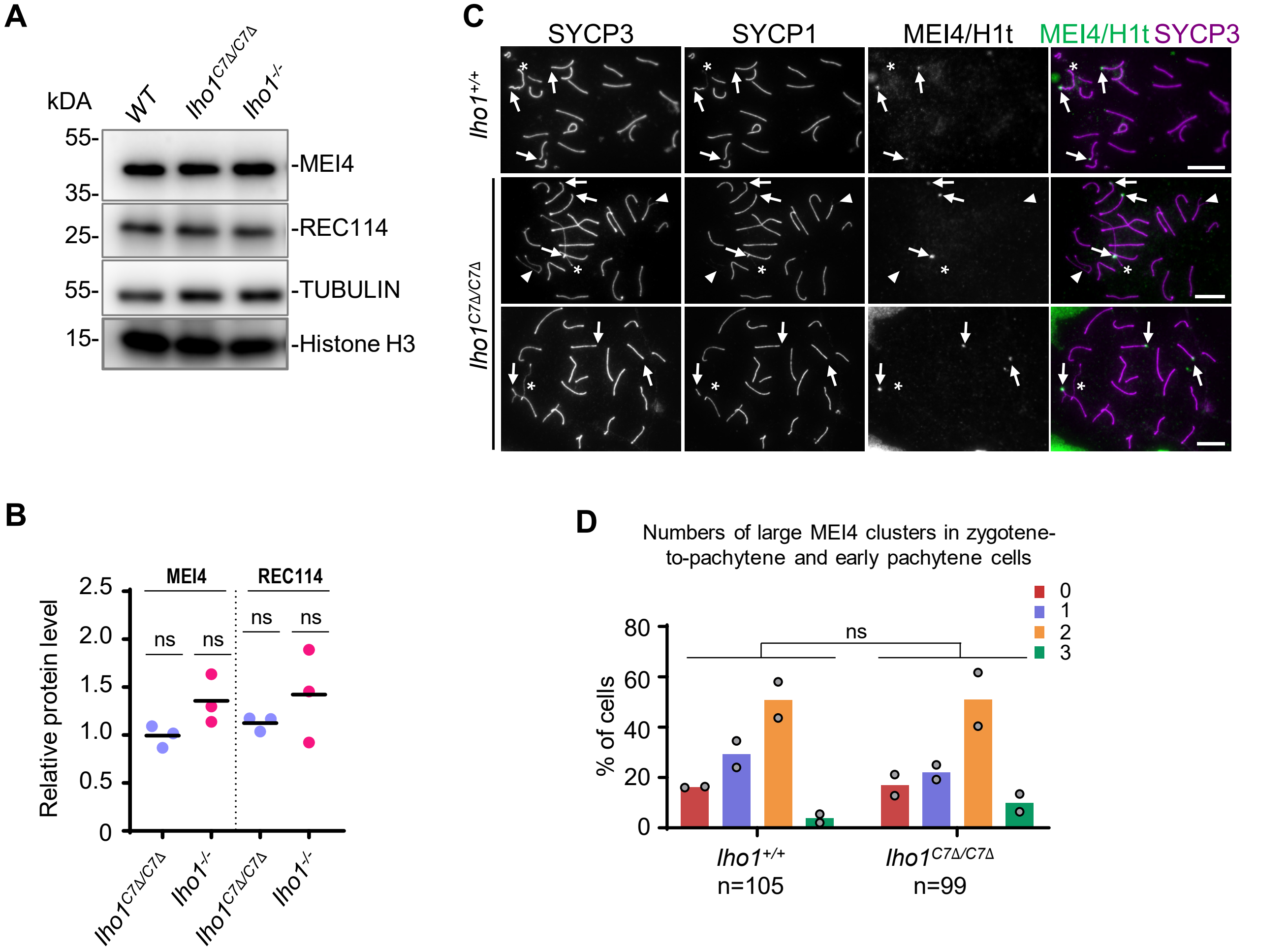

Supplementary Figure 6. MEI4 and REC114 levels and laDSB cluster formation are similar in wild-type and *Iho1*$^{C7\Delta/C7\Delta}$ mice (relevant to Fig. 3)

A Immunoblots of SDS-PAGE of total protein extracts from testes of 13 dpp mice of indicated genotypes. **B** Quantification of total MEI4 and REC114 protein levels in *Iho1*$^{C7\Delta/C7\Delta}$ and *Iho1*$^{-/-}$ mice. Immunoblot signals of MEI4 or REC114 from total testis extracts of 13 dpp *Iho1*$^{C7\Delta/C7\Delta}$ and *Iho1*$^{-/-}$ mice were normalized to corresponding immunoblot signals of 13 dpp wild-type controls. Bar mark means of three experiments; two-tailed one-sample *t* test, ns represents P=0.9343 (*Iho1*$^{C7\Delta/C7\Delta}$, MEI4), 0.135 (*Iho1*$^{-/-}$, MEI4), 0.1073 (*Iho1*$^{C7\Delta/C7\Delta}$, MEI4), 0.2706 (*Iho1*$^{-/-}$, MEI4). **C** Immunostaining of surface spread spermatocytes of adult mice. MEI4 was detected in the same channel as histone H1t, as these two proteins mark distinct populations of spermatocytes, up till early pachytene and beyond mid pachytene, respectively. Panels show correctly synapsed early pachytene cells (*Iho1*$^{+/+}$ and *Iho1*$^{C7\Delta/C7\Delta}$ bottom panel) and an *Iho1*$^{C7\Delta/C7\Delta}$ cell with incomplete synapsis (middle panel) in zygotene-to-pachytene transition or early pachytene-equivalent stage. Arrows, asterisk and arrowheads mark large MEI4 clusters, sex chromosomes, asynaptic autosomes, respectively. Bars, 10 µm. **D** Quantification of MEI4 blob numbers in zygotene-to-pachytene transition and early pachytene spermatocytes of adult mice. Block bars show averages of two experiments, gray circles represent single experiments. Total numbers of counted cells are indicated. Analysis of deviance using the likelihood-ratio test based on the chi-squared distribution, ns represents P=0.2505. Source data are provided as a Source Data file.

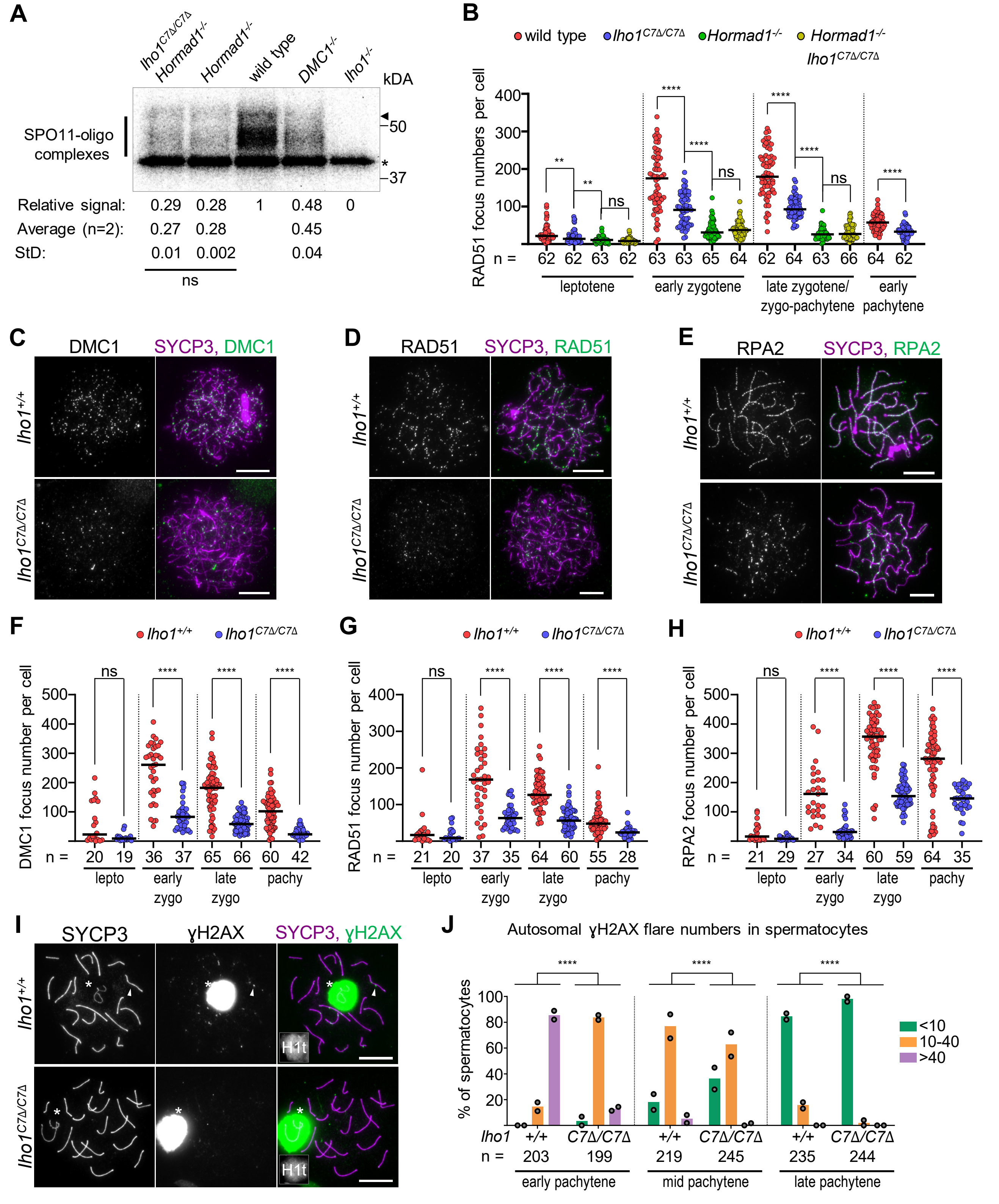

Supplementary Figure 7. Disruption of IHO1-HORMAD1 interaction affects early recombination steps (relevant to Fig. 4)
A Radiograph of immunoprecipitated and radioactively labeled SPO11-oligo complexes from testes of adult mice. Bar, SPO11-specific signals, asterisk, nonspecific labelling, and arrowhead, immunoglobulin heavy-chain. Quantification shows radioactive signals that were background-corrected (*Iho1*$^{-/-}$, signal=0) and normalized to wild type control (1). Means and standard deviations of SPO11-oligo signals are shown from n=2 independent experiments (radiograph shows one of the experiments). Two-tailed paired t-test, ns=P=0.7327. **B** Quantification of axis associated RAD51 focus numbers in spermatocytes. Bars are means, n=cell numbers. Two-tailed Mann Whitney U-test, ns=P>0.05, **=P<0.01, ****=P<0.0001. **C-E, I** Immunostaining of nuclear spread oocytes of 16.5 days-post-coitum (dpc) fetuses (**C-E**) or nuclear spread spermatocytes of adult mice (**I**). Bars, 10 µm. **C-E** Oocytes are shown in early zygotene (**C-D**) or late zygotene (**E**) stages. **I** Spermatocytes are shown in late pachytene stage. Miniaturized images show histone H1t. Sex chromosomes are marked by asterisk. Arrowhead in top panel points at a ɣH2AX flare/foci on a synapsed autosome. **F-H** Quantification of axis associated DMC1 (**F**), RAD51 (**G**) RPA2 (**H**) focus numbers in leptotene (lepto), early zygotene (early zygo), late zygotene (late zygo) and early pachytene (pachy) oocytes of 16.5 dpc fetuses. Bars are medians, n=cell numbers. Two-tailed Mann Whitney U-test, ns=P>0.05, ****=P<0.0001. **J** Quantification of autosomal ɣH2AX flare numbers in spermatocytes of adult mice. Three categories were distinguished, spermatocytes with <10 flares (green), between 10 and 40 flares (orange) and more than 40 flares (lilac) of ɣH2AX on autosomes. Whereas cells with asynaptic chromosomes were also included the quantifications, asynaptic chromosomes exhibit large ɣH2AX domains that are associated with the sex bodies, hence asynaptic chromosomes are not reflected in flare counts. Block bars show averages of two experiments, gray circles represent single experiments, n=number of counted cells. Analysis of deviance using the likelihood-ratio test based on the chi-squared distribution, ****=P<0.0001. Source data, including exact P values, are provided as a Source Data file.

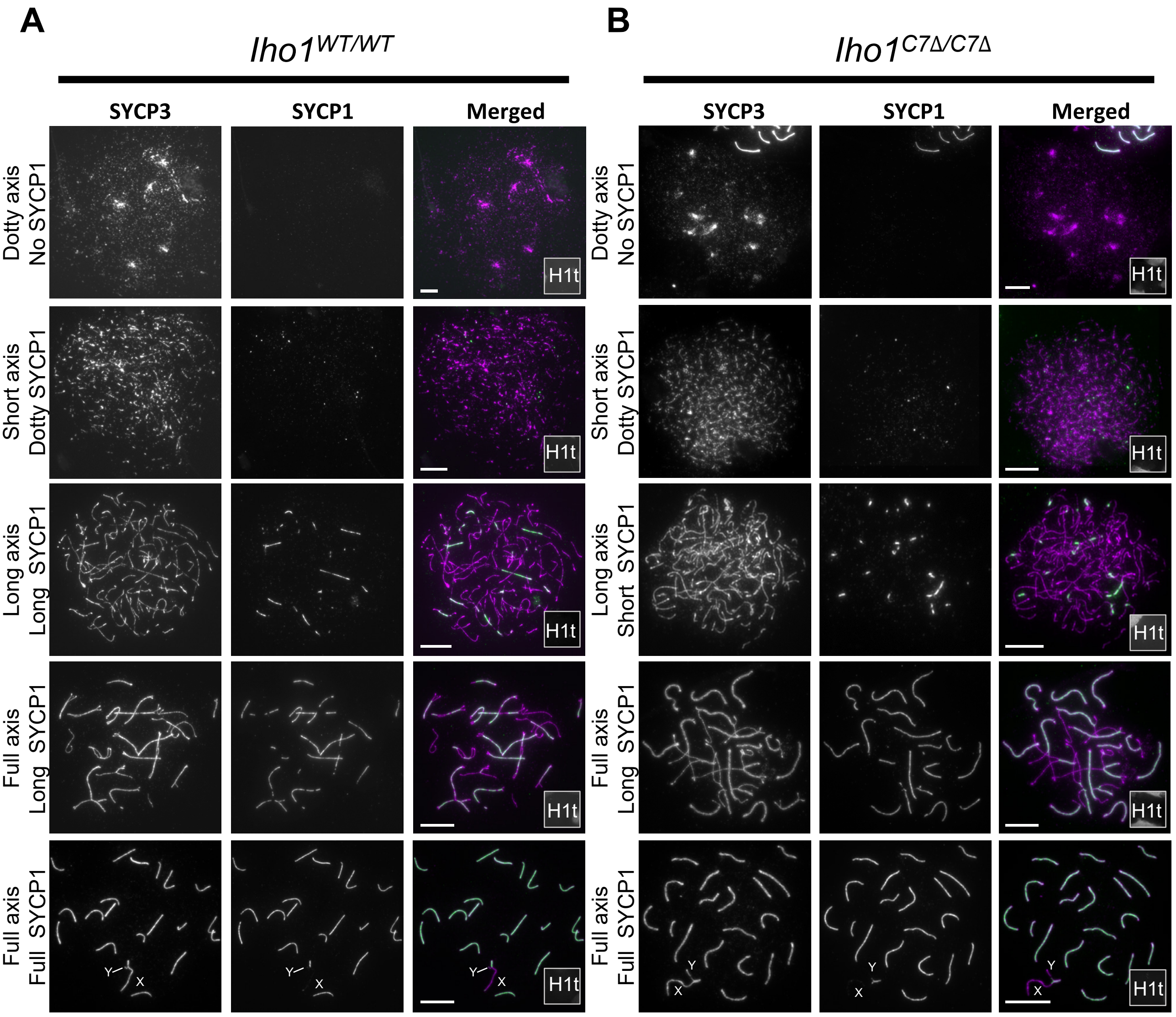

Supplementary Figure 8. Illustration of axis- and SC-morphology categories in wild-type and *Iho1*$^{C7\Delta/C7\Delta}$ spermatocytes (relevant to Fig. 5A)

A-B Immunostaining in nuclear spread spermatocytes of adult mice. Spermatocytes between preleptotene and early pachytene stages are shown. These stages are characterized by an absence of histone H1t (a marker of mid and post mid-pachytene stages). Miniaturized images show histone H1t-specific channel. X and Y chromosomes are indicated in cells where all autosomes have completely formed chromosome axes and SCs. Bars, 10 µm.

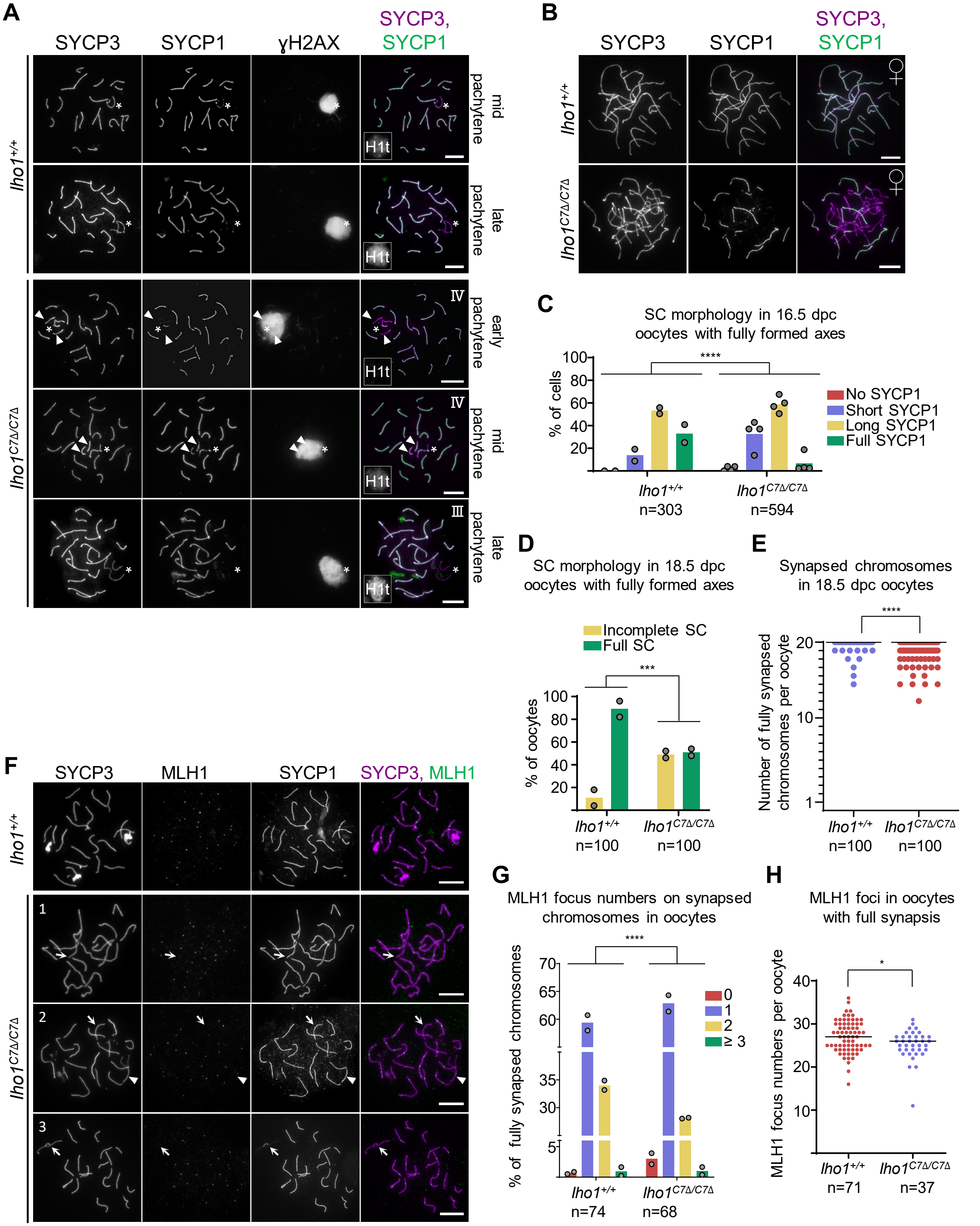

Supplementary Figure 9. SC defects and imperfect CO formation affect both sexes in *Iho1$^{C7\Delta/C7\Delta}$* mice (relevant to Fig. 5)
A-B, F Immunostaining in nuclear spread spermatocytes of adult mice (**A**) or oocytes of 16.5 (**B**) or 18.5 (**C**) dpc fetuses. Bars, 10 µm. **A** Asterisks mark sex chromosomes. Arrowheads mark partially or fully unsynapsed autosomes. Miniaturized images show histone H1t. Roman numbers refer to SC defect types as described in Fig. 5B. **F** Three types of *Iho1$^{C7\Delta/C7\Delta}$* oocytes are shown: (cell 1) all chromosomes are fully synapsed but one chromosome lacks MLH1 (arrow), (cell 2) one chromosome is partially synapsed and have MLH1 focus (arrowhead) and another fully synapsed chromosome lacks MLH1 (arrow), and (cell 3) all synapsed chromosomes have MLH1 foci but one chromosome that is partially synapsed lacks MLH1 focus (arrow). **C-E, G-H** Quantifications of SC morphology in oocytes with fully formed axis at 16.5 dpc (**C**) or 18.5 dpc (**D**), quantification of fully synapsed chromosomes in oocytes from 18.5 dpc fetuses (**E**), and quantifications of MLH1 focus numbers on fully synapsed chromosomes (**G**) or MLH1 focus numbers per cell (**H**) in oocytes of 18.5dpc fetuses. Both fully synapsed and asynaptic (**G**) or only fully synapsed (**H**) pachytene oocytes were examined. Data are pooled from two (**C**, *Iho1$^{+/+}$*, **D-E, G-H**) or four (**C**, *Iho1$^{C7\Delta/C7\Delta}$*) mice. Block bars are means (**C-D, G**), and bars are medians (**E, H**), n=numbers of oocytes. Likelihood ratio test (**C-D, G**) or two-tailed Mann Whitney U-Test (**E, H**), **(C)** ****=P<2.2e-16, **(D)** ***=P=1.45e-4, **(E)** ****=P=8.18e-9, **(G)** ****=P=7.0e-7, **(H)** *=P=0.0197. Source data are provided as a Source Data file.

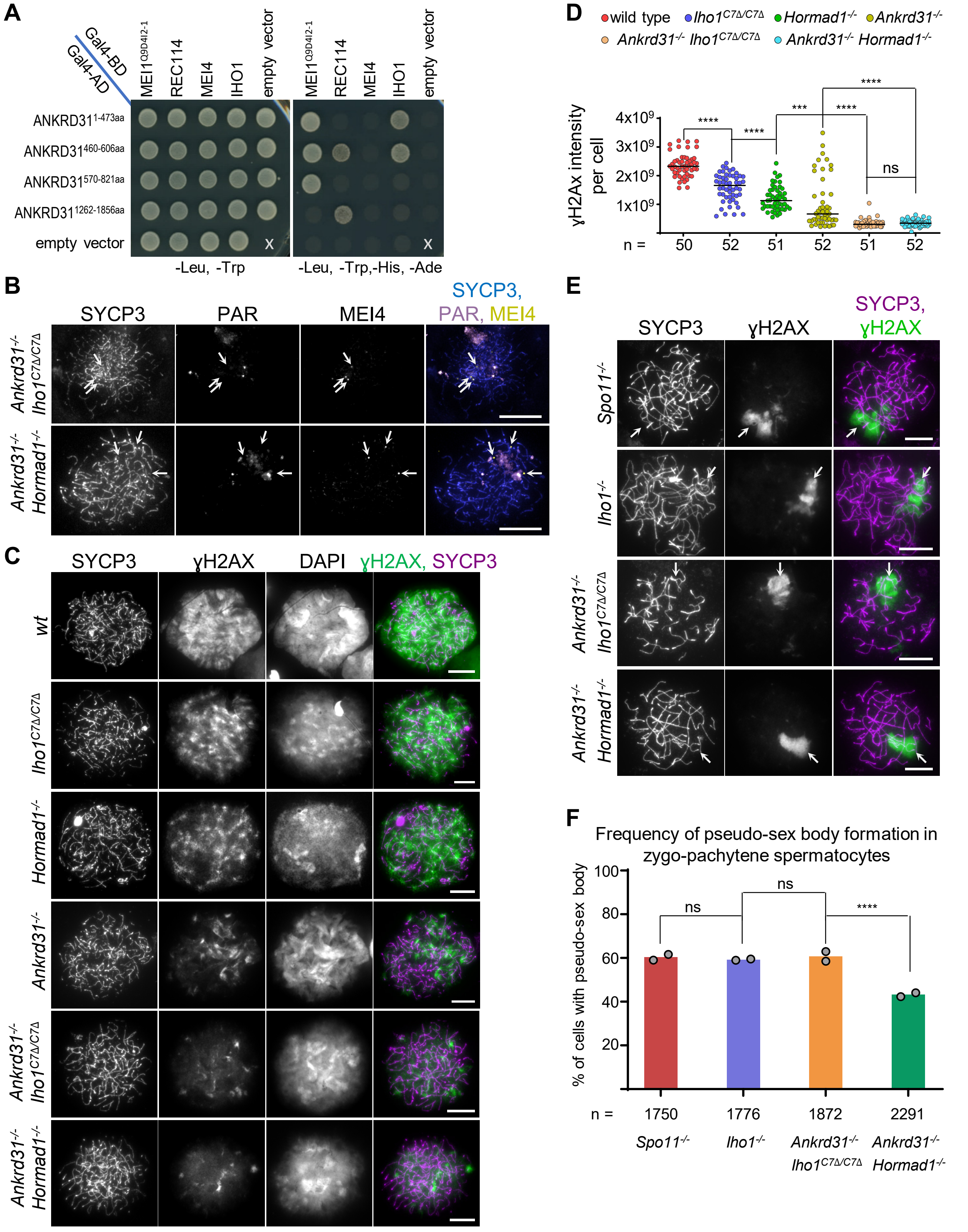

Supplementary Figure 10. Physical and functional interactions of IHO1-HORMAD1 complex and ANKRD31 (relevant to Fig. 7)
A Y2H assays between ANKRD31 fragments and key components of the DSB machinery. Yeast cultures are shown after 2 days of growth on the indicated drop out plates. X marks bait-prey combinations that were omitted from Y2H due to lack of relevance. **B-C, E** Immunostaining (**B-C, E**) and PAR FISH (**B**) in nuclear spread early zygotene (**B-C**, long but incomplete axes) or zygo-pachytene (**E**, fully formed axes) spermatocytes of adult mice**.** Arrows mark sites of MEI4 clusters (**B**) or ɣH2AX-rich chromatin domains, called pseudo-sex bodies (**E**). Bars, 10 µm. **D, F** Quantification of total nuclear ɣH2AX signal (in arbitrary units) in early zygotene spermatocytes (**D**) and quantification of the fraction of zygo-pachytene cells with pseudo-sex body formation (**F**) in adult mice. Bars are medians (**D**), block bars are means (**F**). Datapoints represent cells (**D**) or experiments (**F**). n=number of spermatocytes. Two-tailed Mann Whitney U-Test (**D**) or likelihood ratio test (**F**), ns=P>0.05, ***=P<0.001, ****=P<0.0001. Exact P values: **(D)** wild type vs. *Iho1$^{C7Δ/C7Δ}$* P=2.85e-12, *Iho1$^{C7Δ/C7Δ}$* vs. *Hormad1$^{-/-}$* P=8.33e-5, *Hormad1$^{-/-}$* vs. *Ankrd31$^{-/-}$* P=0.000268, *Ankrd31$^{-/-}$* vs. *Ankrd31$^{-/-}$ Iho1$^{C7Δ/C7Δ}$* P=2.06e-9, *Ankrd31$^{-/-}$* vs. *Ankrd31$^{-/-}$ Hormad1$^{-/-}$* P=2.77e-8, *Ankrd31$^{-/-}$ Iho1$^{C7Δ/C7Δ}$* vs. *Ankrd31$^{-/-}$ Hormad1$^{-/-}$* P=0.1149, **(F)** *Spo11$^{-/-}$* vs. *Iho1$^{-/-}$* P=0.5943, *Spo11$^{-/-}$* vs. *Ankrd31$^{-/-}$ Iho1$^{C7Δ/C7Δ}$* P=0.6535, *Spo11$^{-/-}$* vs. *Ankrd31$^{-/-}$ Hormad1$^{-/-}$* P<2.2e-16, *Iho1$^{-/-}$* vs. *Ankrd31$^{-/-}$ Iho1$^{C7Δ/C7Δ}$* P=0.3211, *Iho1$^{-/-}$* vs. *Ankrd31$^{-/-}$ Hormad1$^{-/-}$* P<2.2e-16, *Ankrd31$^{-/-}$ Iho1$^{C7Δ/C7Δ}$* vs. *Ankrd31$^{-/-}$ Hormad1$^{-/-}$* P<2.2e-16. Source data are provided as a Source Data file.

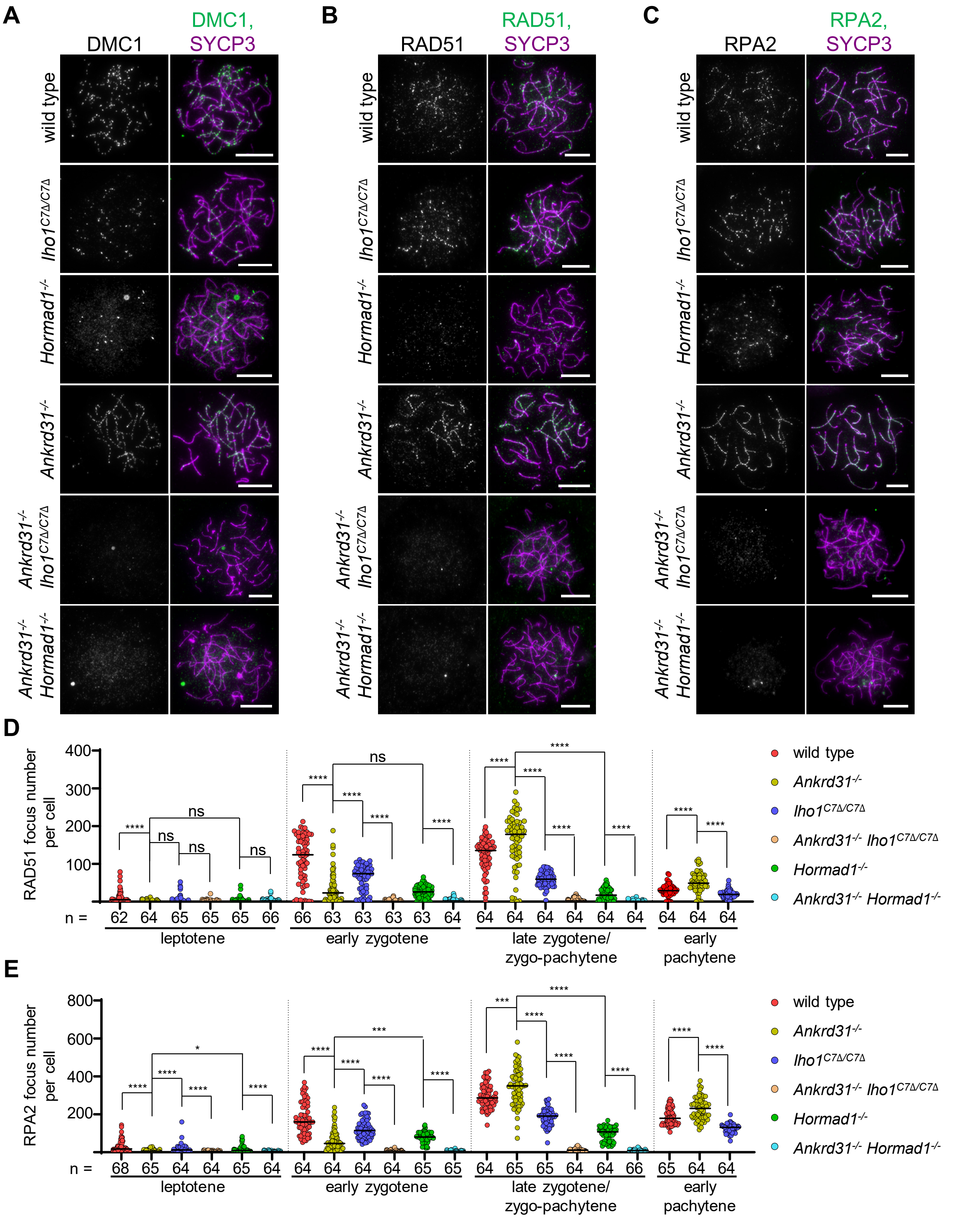

Supplementary Figure 11. Recombination foci are abolished when both IHO1-HORMAD1 complex and ANKRD31 are lost (relevant to Fig. 7)

A-C Immunostaining in nuclear spread late zygotene spermatocytes of adult mice**.** Bars, 10 µm. **D-E** Quantifications of RAD51 (**D**) and RPA2 (**E**) focus numbers in spermatocytes of adult mice. Zygo-pachytene (relevant only to severely SC-defective backgrounds, *Ankrd31*$^{-/-}$ *Iho1*$^{C7\Delta/C7\Delta}$, *Hormad1*$^{-/-}$, *Ankrd31*$^{-/-}$ *Hormad1*$^{-/-}$) is equivalent to a mix of late-zygotene and early pachytene stages which are indistinguishable if SC is defective. Bars are medians, n=cell numbers. Two-tailed Mann Whitney U-Test, ns=P>0.05, *=P< 0.05, ***=P<0.001, ****=P<0.0001. **(D)** (leptotene), wild type vs. *Ankrd31*$^{-/-}$ P=1.46e-5, *Ankrd31*$^{-/-}$ vs. *Iho1*$^{C7\Delta/C7\Delta}$ P=0.6458, *Iho1*$^{C7\Delta/C7\Delta}$ vs. *Ankrd31*$^{-/-}$ *Iho1*$^{C7\Delta/C7\Delta}$ P=0.2106, *Ankrd31*$^{-/-}$ vs. *Hormad1*$^{-/-}$ P=0.304, *Hormad1*$^{-/-}$ vs. *Ankrd31*$^{-/-}$ *Hormad1*$^{-/-}$ P=0.2977, (early zygotene), wild type vs. *Ankrd31*$^{-/-}$ P= 7,88E-10, *Ankrd31*$^{-/-}$ vs. *Iho1*$^{C7\Delta/C7\Delta}$ P= 3,47E-06, *Iho1*$^{C7\Delta/C7\Delta}$ vs. *Ankrd31*$^{-/-}$ *Iho1*$^{C7\Delta/C7\Delta}$ P<2.2e-16, *Ankrd31*$^{-/-}$ vs. *Hormad1*$^{-/-}$ P=0.6998, *Hormad1*$^{-/-}$ vs. *Ankrd31*$^{-/-}$ *Hormad1*$^{-/-}$ P=2.02e-15, (late zygotene), wild type vs. *Ankrd31*$^{-/-}$ P=2,22E-05, *Ankrd31*$^{-/-}$ vs. *Iho1*$^{C7\Delta/C7\Delta}$ P=1,42E-14, *Iho1*$^{C7\Delta/C7\Delta}$ vs. *Ankrd31*$^{-/-}$ *Iho1*$^{C7\Delta/C7\Delta}$ P<2.2e-16, *Ankrd31*$^{-/-}$ vs. *Hormad1*$^{-/-}$ P<2.2e-16, *Hormad1*$^{-/-}$ vs. *Ankrd31*$^{-/-}$ *Hormad1*$^{-/-}$ P=3.72e-12, (early pachytene), wild type vs. *Ankrd31*$^{-/-}$ P=1,76E-08, *Ankrd31*$^{-/-}$ vs. *Iho1*$^{C7\Delta/C7\Delta}$ P=4,83E-14, **(E)** (leptotene), wild type vs. *Ankrd31*$^{-/-}$ P=1,21E-07, *Ankrd31*$^{-/-}$ vs. *Iho1*$^{C7\Delta/C7\Delta}$ P=1,56E-05, *Iho1*$^{C7\Delta/C7\Delta}$ vs. *Ankrd31*$^{-/-}$ *Iho1*$^{C7\Delta/C7\Delta}$ P=6,27E-12, *Ankrd31*$^{-/-}$ vs. *Hormad1*$^{-/-}$ P=0.0206, *Hormad1*$^{-/-}$ vs. *Ankrd31*$^{-/-}$ *Hormad1*$^{-/-}$ P=1,04E-05, (early zygotene), wild type vs. *Ankrd31*$^{-/-}$ P= 7,38E-15, *Ankrd31*$^{-/-}$ vs. *Iho1*$^{C7\Delta/C7\Delta}$ P= 2,05E-10, *Iho1*$^{C7\Delta/C7\Delta}$ vs. *Ankrd31*$^{-/-}$ *Iho1*$^{C7\Delta/C7\Delta}$ P<2.2e-16, *Ankrd31*$^{-/-}$ vs. *Hormad1*$^{-/-}$ P=0,0002151, *Hormad1*$^{-/-}$ vs. *Ankrd31*$^{-/-}$ *Hormad1*$^{-/-}$ P<2.2e-16, (late zygotene), wild type vs. *Ankrd31*$^{-/-}$ P=0,0003343, *Ankrd31*$^{-/-}$ vs. *Iho1*$^{C7\Delta/C7\Delta}$ P<2.2e-16, *Iho1*$^{C7\Delta/C7\Delta}$ vs. *Ankrd31*$^{-/-}$ *Iho1*$^{C7\Delta/C7\Delta}$ P<2.2e-16, *Ankrd31*$^{-/-}$ vs. *Hormad1*$^{-/-}$ P<2.2e-16, *Hormad1*$^{-/-}$ vs. *Ankrd31*$^{-/-}$ *Hormad1*$^{-/-}$ P=9,29E-06, (early pachytene), wild type vs. *Ankrd31*$^{-/-}$ P<2.2e-16, *Ankrd31*$^{-/-}$ vs. *Iho1*$^{C7\Delta}$, P<2.2e-16. Source data are provided as a Source Data file.

Supplementary Table 1. Y2H assays between full length and diverse versions of IHO1 (Gal4AD) and axis components HORMAD1, SYCP2 or SYCP3 (Gal4BD) (relevant to Fig. 1)

Growth of budding yeast in Y2H assay (-Leu, -Trp, -His, -Ade plates) was graded between 0 (no growth) and 3 (strong growth). Grading is not directly comparable in tables S1 and S6. Proteins of interests were tagged on their N terminus with Gal4AD and Gal4BD in most Y2H assays. In addition to N-terminally tagged versions of HORMAD1, C-terminally tagged versions of HORMAD1 were used for testing interactions between some of the IHO1 and HORMAD1 versions. Growth grading is shown separately for N-terminally (**marked by &**) and C-terminally (**marked by #**) tagged HORMAD1 (full lengths or fragments) if Y2H interactions were affected by the site of HORMAD1 tagging. Y2H assays are **marked by *** if interaction tests between IHO1 and N-terminally or C-terminally tagged HORMAD1 resulted in similar yeast growth. SYCP2 fragment (1200-1500)-GAL4BD (**marked by §**) autoactivates, leading to growth after three days; 0 grading refers to no additional growth relative to autoactivation in this case. Amino acid positions of the beginning and the end of protein fragments are indicated. Bold fragments of IHO1 contained a coiled coil domain, as predicted by alphafold (amino acid positions 114-246). In mutated forms of IHO1, we indicate the positions and mutations of the serines and the threonine that are phosphorylated in vivo. HORMAD1 fragment 17-239 contains the full length of HORMA domain (amino acid positions 25-227). n. t.=not tested.

		HORMAD1						SYCP3	SYCP2								
		full length 1-392	1-107	17-239	108-304	240-392	305-392	full length 1-254	1-549	1-390	1-276	270-390	390-1300	390-800	800-1300	1200-1500§
IHO1 fragments (amino acid positions are indicated)	1-574 (full length wild type form)	**3***	**$0^{\&}$**	**3***	**$0^{\&}$**	**$0^{\&}$**	**$0^{\&}$**	**0**	**0**	**0**	**0**	**0**	**0**	**0**	**0**	**0**
	1-227	**0***	not tested	not tested	not tested	not tested	not tested	not tested	not tested	not tested	not tested	not tested	not tested	not tested	not tested	not tested
	**1-358**	**0***	not tested	not tested	not tested	not tested	not tested	not tested	not tested	not tested	not tested	not tested	not tested	not tested	not tested	not tested
	**1-567 (C7Δ)**	**0***	**$0^{\&}$**	**0***	**$0^{\&}$**	**$0^{\&}$**	**$0^{\&}$**	**0**	**0**	**0**	**0**	**0**	**0**	**0**	**0**	**0**
	**105-364**	**$0^{\&}$**	not tested	not tested	not tested	not tested	not tested	not tested	not tested	not tested	not tested	not tested	not tested	not tested	not tested	not tested
	197-455	**$0^{\&}$**	not tested	not tested	not tested	not tested	not tested	not tested	not tested	not tested	not tested	not tested	not tested	not tested	not tested	not tested
	351-514	**$0^{\&}$**	not tested	not tested	not tested	not tested	not tested	not tested	not tested	not tested	not tested	not tested	not tested	not tested	not tested	not tested
	228-574	**$3^{\&}$**	not tested	not tested	not tested	not tested	not tested	not tested	not tested	not tested	not tested	not tested	not tested	not tested	not tested	not tested
	359-574	**3***	not tested	**3***	not tested	not tested	not tested	not tested	not tested	not tested	not tested	not tested	not tested	not tested	not tested	not tested
	440-574	**$2^{\#}/0^{\&}$**	not tested	**$3^{\#}/2^{\&}$**	not tested	not tested	not tested	not tested	not tested	not tested	not tested	not tested	not tested	not tested	not tested	not tested
	500-574	**$1^{\#}/0^{\&}$**	not tested	**$2^{\#}/1^{\&}$**	not tested	not tested	not tested	not tested	not tested	not tested	not tested	not tested	not tested	not tested	not tested	not tested
	**full length with mutation: 476A-490A-569S-570S**	**$3^{\&}$**	not tested	not tested	not tested	not tested	not tested	not tested	not tested	not tested	not tested	not tested	not tested	not tested	not tested	not tested
	**full length with mutation: 476S-490T-569A-570A**	**0***	**$0^{\&}$**	**$0^{\&}$**	**$0^{\&}$**	**$0^{\&}$**	**$0^{\&}$**	not tested	not tested	not tested	not tested	not tested	not tested	not tested	not tested	not tested
	**full length with mutation:476S-490T-569D-570D**	**$0^{\&}$**	not tested	not tested	not tested	not tested	not tested	not tested	not tested	not tested	not tested	not tested	not tested	not tested	not tested	not tested
	**full length with mutation:476S-490T-569E-570E**	**$0^{\&}$**	not tested	not tested	not tested	not tested	not tested	not tested	not tested	not tested	not tested	not tested	not tested	not tested	not tested	not tested
	**full length with mutation:476S-490T-569S-570A**	**$2.5^{\&}$**	not tested	not tested	not tested	not tested	not tested	not tested	not tested	not tested	not tested	not tested	not tested	not tested	not tested	not tested
	**full length with mutation:476S-490T-569S-570D**	**$3^{\&}$**	not tested	not tested	not tested	not tested	not tested	not tested	not tested	not tested	not tested	not tested	not tested	not tested	not tested	not tested
	**full length with mutation:476S-490T-569S-570E**	**$3^{\&}$**	not tested	not tested	not tested	not tested	not tested	not tested	not tested	not tested	not tested	not tested	not tested	not tested	not tested	not tested
	**full length with mutation:476S-490T-569A-570S**	**$0^{\&}$**	not tested	not tested	not tested	not tested	not tested	not tested	not tested	not tested	not tested	not tested	not tested	not tested	not tested	not tested
	**full length with mutation:476S-490T-569D-570S**	**$1.5^{\&}$**	not tested	not tested	not tested	not tested	not tested	not tested	not tested	not tested	not tested	not tested	not tested	not tested	not tested	not tested
	**full length with mutation:476S-490T-569E-570S**	**$1^{\&}$**	not tested	not tested	not tested	not tested	not tested	not tested	not tested	not tested	not tested	not tested	not tested	not tested	not tested	not tested

Supplementary Table 2. Summary of molecular features underlying the interactions between Mer2/IHO1- and HORMAD1-family proteins and the reported roles of the C-terminal region of Mer2/IHO1-family proteins in diverse taxa according to the present study and prior publications [10-13] (relevant to Fig. 1)

The lengths of proteins are indicated in brackets, and amino acid (aa) positions are indicated for motifs/domains, interacting regions and mutations. Amino acid positions for predicted domains/motifs are shown according to publications in the cases of (i) coiled coil in IHO1/Mer2 family [14], (ii) SSM2 in IHO1/Mer2 family [10], (iii) acidic patch in IHO1/Mer2 family (this study), (iv) HORMA domain and PHD domain in budding yeast [15] or according to Uniprot database in the cases of HORMA, PHD and SWIRM domains in mouse, *Arabidopsis thaliana* , and fission yeast. If the table indicates that a region is required for interaction, the requirement was tested with otherwise full-length proteins.

species	molecular requirements for interactions between Mer2/IHO1- and HORMAD1-family proteins											mutations in the C-terminus of Mer2/IHO1-family proteins examined *in vivo*			additional notes	references
	IHO1/ Mer2 family	positions of relevant domains/motifs			HORMAD1 family	positions of relevant domains/motifs			regions of HORMAD1-family proteins interacting IHO1/Mer2	regions in IHO1/Mer2 family affecting interaction		mutations	lost interactions of Mer2/IHO1-family proteins	phenotypes		
		coiled coil	SSM2	acidic patch		HORMA	PHD	SWIRM		positions of regions	role in interaction					
mouse	IHO1 (574)	109-267	556-571	566-572 DFDSSDD	HORMAD1 (392)	25-227	no	no	full length and 17-239 (HORMA domain)	500-574 (SSM2-acidic patch)	sufficient	deletion of aa 568-574 (acidic patch)	HORMAD1	reduced DSB-factor accumulation on axes, reduced number of DSBs (~50% of wild type)	none	this study
										567-574 (SSM2-acidic patch)	required					
Arabidopsis thaliana	PRD3 (449)	120-270	389-404	397-404 DSDEEDID	ASY1(596)	15-228	no	351-449	306-596 (SWIRM)	1-261 (coiled coil)	sufficient and required	not reported			interaction detected at high stringency	Vrielynck et al., 2021 [11]
									1-300 (HORMA domain)	397-404 (acidic patch)	required				interaction detected only at low stringency	this study
budding yeast	Mer2 (314)	41-224	294-304	303-314 EELRPDTLESEL	Hop1 (605)	22-244	329-526	no	full length and requirement for 16-246 (HORMA domain)	140-256 (coiled coil)	sufficient	not reported			none	Rousova et al., 2021 [12]
										256-314 (SSM2-acidic patch)	not required if 1-139 of Mer2 is absent					
											required if 1-139 is present next to 140-256 in Mer2					
fission yeast	Rec15 (180)	1-160	164-180	173-179 DFLADDD	Hop1 (528)	11-212	334-385	no	301-527 (PHD)	161-180 (SSM2-acidic patch)	required	deletion of aa 161-180 (SSM2 and acidic patch)	Rec10 (core axis component, an ortholog of mammalian SYCP2/3) and Hop1	binding of DSB hotspot regions by axis component Rec10 is lost, binding of axis sites by Rec15 is lost, low spore viability and absence of recombination; whereas DSB numbers were not reported, the recombination defects phenocopy Rec10 loss, which results in complete loss of DSBs	Rec10 loss or deletion of aa 161-180 in Rec15 cause more severe phenotypes than Hop1 loss. DSBs form at approximately 40% of wild type levels, and spore viability is high in the absense of Hop1, indicating that DSBs are more reliant on Rec15-Rec10 than Rec15-Hop1 interactions.	Kariyazono et al., 2019 [13]
Sordaria macrospora	Mer2 (504)	55-275	480-495	not present	not reported				not applicable			deletion aa 450-504 (preSSM2 and SSM2)	not reported	no DSBs; phenotypes that cannot be explained by DSB role: (i) delayed chromosome condensation in late meiotic prophase and (ii) mutant protein fails to accumulate on chromatin possibly due to a failure to enter nucleus	Both deletion 450-504 and Q461R cause complete loss of DSBs, which suggest that preSSM2 region is important for DSB formation. SSM2 is also involved in post-DSB functions, as the SSM2 mutation (LG481/2PW) barely affects DSBs but disrupts post-DSB features of meiosis. Consistent with a role post-DSB formation, *Sm* Mer2 is not depleted from synapsed chromatin, and *Sm* Mer2 persist on chromatin even beyond meiotic divisions. This feature is unique among examined taxa.	Tesse et al., 2017 [10]
												substitution: Q461R (change in preSSM2)		no DSBs; phenotypes that cannot be explained by DSB role: delayed chromosome condensation in late meiotic prophase		
												substitutions: LG481/2PW (change in SSM2)		nearly normal DSB numbers; phenotypes that cannot be explained by DSB role: (i) delayed chromosome alignment and (ii) altered CO patterning in early and mid prophase, and (iii) delayed chromosome condensation in late meiotic prophase, which correlates with (iv) premature depletion of Mer2 from chromosomes in late prophase		

Supplementary Table 3. MS analysis of in vitro phosphorylated C-terminal peptides of IHO1 (relevant to Fig. 2)

Calculated fragments of NLLCDPDFDS(pS)DNF-COOH and –NH2 peptides corresponding to the MS/MS spectra shown in Fig. 2D and Supplementary Fig. 3E, respectively. Identified b- and y-ions are shown in red and blue, respectively. b- and y-ions with neutral losses of phosphorylation, H2O and NH3 are also indicated in the table.

NLLCDPDFDS(pS)DDNF-COOH corresponding to Fig. 2D

Fragments and Phosphorylation losses (yellow)

#1	b^+-Phos	b^{2+}-Phos	b^+	b^{2+}	b^{3+}	Seq.	y^+	y^{2+}	y^{3+}	y^+-Phos	y^{2+}-Phos	#2
1			115.05020	58.02874	39.02159	N						15
2			228.13427	114.57077	76.71627	L	1682.59277	841.80003	561.53578	1584.61588	792.81158	14
3			341.21833	171.11280	114.41096	L	1569.50871	785.25799	523.84109	1471.53182	736.26955	13
4			444.22752	222.61740	148.74736	C	1456.42465	728.71596	486.14640	1358.44775	679.72751	12
5			559.25446	280.13087	187.08967	D	1353.41546	677.21137	451.81001	1255.43857	628.22292	11
6			656.30722	328.65725	219.44059	P	1238.38852	619.69790	413.46769	1140.41162	570.70945	10
7			771.33417	386.17072	257.78291	D	1141.33576	571.17152	381.11677	1043.35886	522.18307	9
8			918.40258	459.70493	306.80571	F	1026.30881	513.65804	342.77446	928.33192	464.66960	8
9			1033.42952	517.21840	345.14803	D	879.24040	440.12384	293.75165	781.26350	391.13539	7
10			1120.46155	560.73441	374.15870	S	764.21346	382.61037	255.40934	666.23656	333.62192	6
11	1189.48302	595.24515	1287.45991	644.23359	429.82482	S-Phospho	677.18143	339.09435	226.39866	579.20453	290.10590	5
12	1304.50996	652.75862	1402.48685	701.74707	468.16714	D	510.18307	255.59517	170.73254			4
13	1419.53690	710.27209	1517.51380	759.26054	506.50945	D	395.15613	198.08170	132.39023			3
14	1533.57983	767.29355	1631.55672	816.28200	544.52376	N	280.12918	140.56823	94.04791			2
15						F	166.08626	83.54677	56.03360			1

Fragments with neutral losses of H2O and NH3

#1	b-H_2O^+	b-H_2O^{2+}	b-H_2O^{3+}	b-NH_3^+	b-NH_3^{2+}	b-NH_3^{3+}	Seq.	y-H_2O^+	y-H_2O^{2+}	y-H_2O^{3+}	y-NH_3^+	y-NH_3^{2+}	y-NH_3^{3+}	#2
1				98.02365	49.51547	33.34607	N							15
2				211.10772	106.05750	71.04076	L	1664.58221	832.79474	555.53225	1665.56623	833.28675	555.86026	14
3				324.19178	162.59953	108.73545	L	1551.49815	776.25271	517.83757	1552.48216	776.74472	518.16557	13
4				427.20097	214.10412	143.07184	C	1438.41408	719.71068	480.14288	1439.39810	720.20269	480.47088	12
5	541.24389	271.12559	181.08615	542.22791	271.61759	181.41415	D	1335.40490	668.20609	445.80648	1336.38891	668.69810	446.13449	11
6	638.29666	319.65197	213.43707	639.28067	320.14398	213.76508	P	1220.37796	610.69262	407.46417	1221.36197	611.18462	407.79217	10
7	753.32360	377.16544	251.77938	754.30762	377.65745	252.10739	D	1123.32519	562.16623	375.11325	1124.30921	562.65824	375.44125	9
8	900.39202	450.69965	300.80219	901.37603	451.19165	301.13019	F	1008.29825	504.65276	336.77093	1009.28226	505.14477	337.09894	8
9	1015.41896	508.21312	339.14450	1016.40297	508.70513	339.47251	D	861.22983	431.11856	287.74813	862.21385	431.61056	288.07613	7
10	1102.45099	551.72913	368.15518	1103.43500	552.22114	368.48319	S	746.20289	373.60508	249.40581	747.18691	374.09709	249.73382	6
11	1269.44935	635.22831	423.82130	1270.43336	635.72032	424.14930	S-Phospho	659.17086	330.08907	220.39514	660.15488	330.58108	220.72314	5
12	1384.47629	692.74178	462.16361	1385.46030	693.23379	462.49162	D	492.17250	246.58989	164.72902	493.15652	247.08190	165.05702	4
13	1499.50323	750.25525	500.50593	1500.48725	750.74726	500.83393	D	377.14556	189.07642	126.38670	378.12958	189.56843	126.71471	3
14	1613.54616	807.27672	538.52024	1614.53017	807.76873	538.84824	N				263.10263	132.05496	88.37240	2
15							F							1

NLLCDPDFDS(pS)DDNF-NH2 corresponding to Fig. S2D

Fragments and Phosphorylation losses (yellow)

#1	b^+-Phos	b^{2+}-Phos	b^+	b^{2+}	b^{3+}	Seq.	y^+	y^{2+}	y^{3+}	y^+-Phos	y^{2+}-Phos	#2		
1			115.05020	58.02874	39.02159	N						15		
2			228.13427	114.57077	76.71627	L	1681.60876	841.30802	561.20777	1583.63186	792.31957	14		
3			341.21833	171.11280	114.41096	L	1568.52470	784.76599	523.51308	1470.54780	735.77754	13		
4			444.22752	222.61740	148.74736	C	1455.44063	728.22395	485.81839	1357.46374	679.23551	12		
5			559.25446	280.13087	187.08967	D	1352.43145	676.71936	451.48200	1254.45455	627.73091	11		
6			656.30722	328.65725	219.44059	P	1237.40450	619.20589	413.13969	1139.42761	570.21744	10		
7			771.33417	386.17072	257.78291	D	1140.35174	570.67951	380.78876	1042.37484	521.69106	9		
8			918.40258	459.70493	306.80571	F	1025.32480	513.16604	342.44645	927.34790	464.17759	8		
9			1033.42952	517.21840	345.14803	D	878.25638	439.63183	293.42325	780.27949	390.64338	7		
10			1120.46155	560.73441	374.15870	S	763.22944	382.11836	255.08133	665.25255	333.12991	6		
11	1189.48302	595.24515	1287.45991	644.23359	429.82482	S-Phospho	676.19741	338.60234	226.07066	578.22052	289.61390	5		
12	1304.50996	652.75862	1402.48685	701.74707	468.16714	D	509.19905	255.10316	170.40454			4		
13	1419.53690	710.27209	1517.51380	759.26054	506.50945	D	394.17211	197.58969	132.06222			3		
14	1533.57983	767.29355	1631.55672	816.28200	544.52376	N	279.14517	140.07622	93.71991			2		
15						F-Amidated	165.10224	83.05476	55.70560			1		

Fragments with neutral losses of H2O and NH3

#1	b-H_2O^+	b-H_2O^{2+}	b-H_2O^{3+}	b-NH_3^+	b-NH_3^{2+}	b-NH_3^{3+}	Seq.	y-H_2O^+	y-H_2O^{2+}	y-H_2O^{3+}	y-NH_3^+	y-NH_3^{2+}	y-NH_3^{3+}	#2
1				98.02365	49.51547	33.34607	N							15
2				211.10772	106.05750	71.04076	L	1663.59819	832.30274	555.20425	1664.58221	832.79474	555.53225	14
3				324.19178	162.59953	108.73545	L	1550.51413	775.76070	517.50956	1551.49815	776.25271	517.83757	13
4				427.20097	214.10412	143.07184	C	1437.43007	719.21867	479.81487	1438.41408	719.71068	480.14288	12
5	541.24389	271.12559	181.08615	542.22791	271.61759	181.41415	D	1334.42088	667.71408	445.47848	1335.40490	668.20609	445.80648	11
6	638.29666	319.65197	213.43707	639.28067	320.14398	213.76508	P	1219.39394	610.20061	407.13616	1220.37795	610.69262	407.46417	10
7	753.32360	377.16544	251.77938	754.30762	377.65745	252.10739	D	1122.34118	561.67423	374.78524	1123.32519	562.16623	375.11325	9
8	900.39202	450.69965	300.80219	901.37603	451.19165	301.13019	F	1007.31423	504.16075	336.44293	1008.29825	504.65276	336.77093	8
9	1015.41896	508.21312	339.14450	1016.40297	508.70513	339.47251	D	860.24582	430.62655	287.42012	861.22983	431.11856	287.74813	7
10	1102.45099	551.72913	368.15518	1103.43500	552.22114	368.48319	S	745.21888	373.11308	249.07781	746.20289	373.60508	249.40581	6
11	1269.44935	635.22831	423.82130	1270.43336	635.72032	424.14930	S-Phospho	658.18685	329.59706	220.06713	659.17086	330.08907	220.39514	5
12	1384.47629	692.74178	462.16361	1385.46030	693.23379	462.49162	D	491.18849	246.09788	164.40101	492.17250	246.58989	164.72902	4
13	1499.50323	750.25525	500.50593	1500.48725	750.74726	500.83393	D	376.16154	188.58441	126.05870	377.14556	189.07642	126.38670	3
14	1613.54616	807.27672	538.52024	1614.53017	807.76873	538.84824	N				262.11862	131.56295	88.04439	2
15							F-Amidated							1

Supplementary Table 4. Fertility checks in *Iho1* $^{C7\Delta/C7\Delta}$ mice (relevant to Fig. 3)

Quantification of pup numbers from crosses of wild type to wild type (wt) and wild type to Iho1C7Δ/C7Δ mice. Statistical significance was calculated by two tailed t-test with Welch correction.

female age			female < 28 weeks		female >28 weeks	
female	wt		wt	*Iho1* $^{C7\Delta/C7\Delta}$	wt	*Iho1* $^{C7\Delta/C7\Delta}$
male	wt	*Iho1* $^{C7\Delta/C7\Delta}$	wt			
breeding pairs	5	5	9	12	9	12
pups/breeding week	1,231	1,426	1,331	1,205	1,168	0,8285
total breeding weeks	378	416	175	234	151	206
Significance, P=	0,3584		0,1356		0,0106	

Supplementary Table 5. Spermatocyte apoptosis in *Iho1* $^{C7\Delta/C7\Delta}$ mice (relevant to Fig. 3)

Quantification of seminiferous tubules where apoptosis was detected in the cell layer consisting of pachytene, diplotene or meiotically dividing cells. Stages of the epithelial seminiferous cycle are indicated. Apoptosis was detected by immunostaining for cleaved PARP.

	tubule stages	no apoptotic cell		1-4 apoptotic cells		5-15 apoptotic cells		sum of tubules		sum of apoptotic tubules		% of apoptotic tubules		
		mouse 1	mouse 2	mouse 1	mouse 2	mouse 1	mouse 2	mouse 1	mouse 2	mouse 1	mouse 2	mouse 1	mouse 2	mean %
wild-type mice	Stage I	9	5	1	0	0	0	10	5	1	0	10,0%	0,0%	5,0%
	Stage II-III	8	9	0	2	0	0	8	11	0	2	0,0%	18,2%	9,1%
	Stage IV	9	14	2	0	0	0	11	14	2	0	18,2%	0,0%	9,1%
	Stage V-VI	11	29	2	7	0	0	13	36	2	7	15,4%	19,4%	17,4%
	Stage VII-VIII	28	20	2	3	0	0	30	23	2	3	6,7%	13,0%	9,9%
	Stage IX	14	14	1	5	0	0	15	19	1	5	6,7%	26,3%	16,5%
	Stage X-XI	23	17	3	1	0	0	26	18	3	1	11,5%	5,6%	8,5%
	Stage XII	7	11	2	2	0	0	9	13	2	2	22,2%	15,4%	18,8%
	all stages	109	119	13	20	0	0	122	139	13	20	10,7%	14,4%	12,5%
Iho1 $^{C7\Delta/C7\Delta}$ mice	Stage I	8	13	2	2	0	1	10	16	2	3	20,0%	18,8%	19,4%
	Stage II-III	9	13	0	0	0	0	9	13	0	0	0,0%	0,0%	0,0%
	Stage IV	6	17	4	6	1	1	11	24	5	7	45,5%	29,2%	37,3%
	Stage V-VI	1	9	5	14	6	3	12	26	11	17	91,7%	65,4%	78,5%
	Stage VII-VIII	4	6	4	24	8	6	16	36	12	30	75,0%	83,3%	79,2%
	Stage IX	7	7	3	10	0	1	10	18	3	11	30,0%	61,1%	45,6%
	Stage X-XI	7	15	9	15	0	1	16	31	9	16	56,3%	51,6%	53,9%
	Stage XII	2	7	9	7	1	6	12	20	10	13	83,3%	65,0%	74,2%
	all stages	44	87	36	78	16	19	96	184	52	97	54,2%	52,7%	53,4%

Supplementary Table 6. Y2H interactions between ANKRD31 fragments (Gal4 AD) and key components of the DSB machinery (Gal4 BD) (relevant to Fig. 7)

Growth of budding yeast in Y2H assay (-Leu, -Trp, -His, -Ade plates) was graded between 0 (no growth) and 4 (very strong growth). Bold fragments 376-821 and 460-606 contained ankyrin repeats 1-3 (amino acid positions 475-570) and fragment 1055-1459 contained ankyrin repeats 4-6 (amino acid positions 1162-1257). * IHO1-GAL4BD fusion mildly autoactivates leading to weak growth beyond three days, hence IHO1 interactions were scored after two days of growth on selective media. n. t.=not tested.

		Tested components of the DSB machinery							
		HORMAD1	MEI1	REC114	MEI4	IHO1 *	SPO11 alpha form	SPO11 beta form	TOPVIBL
ANNKRD31 fragments (amino acid positions are indicated)	1-473	0	4	0	0	3	0	0	0
	**376-821**	0	4	3	0	4	0	0	0.5
	**460-606**	not tested	4	3	0	3	not tested	not tested	0.5
	570-821	not tested	4	0	0	0	not tested	not tested	0
	750-1150	0	0	0	0	0	0	0	0
	**1055-1459**	0	0	0	0	0	0	0	0
	1262-1857	0	0	3.5	0	0	0	0	0
	1260-1460	not tested	not tested	0	not tested	not tested	not tested	not tested	not tested
	1460-1790	not tested	not tested	0	not tested	not tested	not tested	not tested	not tested
	1784-1857	not tested	not tested	2	not tested	not tested	not tested	not tested	not tested

Supplementary Table 7.

REAGENTS AND RESOURCES

REAGENT or RESOURCE	SOURCE	IDENTIFIER	Dilution
Antibodies			
Rabbit polyclonal anti-GFP	Thermo Fisher Scientific	Cat# A-11122<br>RRID: AB_221569	1:200 for IF
Chicken polyclonal anti-SYCP3	[16]	N/A	1:100 for IF<br>1:1000 for WB
Mouse monoclonal anti-SYCP3	[17]	N/A	1:1 for IF
Guinea pig polyclonal anti-IHO1	[4]	N/A	1:300 for IF<br>1:1000 for WB
Rabbit polyclonal anti-IHO1	[4]	N/A	1:1000 for IF<br>1:3000 for WB
Guinea pig polyclonal anti-HORMAD1	[9]	N/A	1:400 for IF<br>1:1500 for WB
Rabbit polyclonal anti-HORMAD1	[9]	N/A	1:700 for IF<br>1:1500 for WB
Guinea pig polyclonal anti-MEI4	[4]	N/A	1:300 for IF<br>1:1000 for WB
Rabbit polyclonal anti-REC114	[2]	N/A	1:500 for IF<br>1:1500 for WB
Rabbit polyclonal anti-SYCP3	Abcam	Cat# ab15093<br>RRID: AB_301639	1:1000 for IF<br>1:2000 for WB
Mouse monoclonal anti- β-Actin	Santa Cruz	Cat# ab47778<br>RRID: AB_626632	1:1000 for WB
Mouse monoclonal anti-GAPDH	Santa Cruz	Cat# sc-32233<br>RRID: AB_627679	1:1000 for WB
Rabbit polyclonal anti-Histone H3	Abcam	Cat# ab1791<br>RRID: AB_302613	1:100000 for WB
Mouse monoclonal anti-α-TUBULIN	Sigma-Aldrich	Cat# T6199<br>RRID: AB_477583	1:3000 for WB

Rabbit polyclonal anti-DMC1 (H-100)	Santa Cruz	Cat# sc-22768;<br>RRID: AB_2277191	1:100 for IF
Rabbit polyclonal anti-Rad51 (H-92)	Santa Cruz	Cat# sc-8349;<br>RRID: AB_2253533	1:100 for IF
Rat monoclonal anti-RPA32 (4E4)	Cell signaling	Cat# 2208;<br>RRID: AB_2238543	1:100 for IF
Rabbit polyclonal anti-cleaved PARP (Asp214)	Cell signaling	Cat# 9544;<br>RRID: AB_2160724	1:500 for IF
Mouse monoclonal anti-phospho-Histone H2A.X (Ser139)	Millipore	Cat# 05-636;<br>RRID: AB_309864	1:3000 for IF
Rabbit polyclonal anti-MVH	Abcam	Cat# ab13840<br>RRID: AB_443012	1:500 for IF
Mouse monoclonal anti-p63	Biocare Medical	Cat# CM163A<br>RRID: AB_10582730	1:200 for IF
Guinea pig polyclonal anti-Histone H1t	[2]	N/A	1:5000 for IF
Rabbit polyclonal anti-Histone H1t	[2]	N/A	1:20000 for IF
Mouse monoclonal anti-SPO11(Spo11-180)	MSKCC Antibody and Bioresource Core Facility	N/A	1:2000 for IP
Chicken polyclonal anti-SYCP1	[2]	N/A	1:300 for IF
Rabbit polyclonal anti-SYCP1	Abcam	Cat# ab15090<br>RRID: AB_301636	1:1000 for IF
Mouse monoclonal anti-MLH1	Cell signaling	Cat# 3515S;<br>RRID: AB_2145615	1:100 for IF
Goat anti-rabbit IgG-HRP	Jackson ImmunoResearch	Cat# 111-035-003; RRID: AB_2313567	1:10000 for WB
Goat anti-guinea pig IgG-HRP	Jackson ImmunoResearch	Cat# 706-035-148; RRID: AB_2340447	1:10000 for WB
Goat anti-mouse IgG-HRP	Jackson ImmunoResearch	Cat# 115-035-003; RRID: AB_10015289	1:10000 for WB
Goat anti-Rabbit IgG-AF405	Thermo Fisher Scientific	Cat# A-31556; RRID: AB_221605	1:200 for IF
Goat anti-Rabbit IgG-AF488	Thermo Fisher Scientific	Cat# A-11034; RRID: AB_2576217	1:600 for IF

Goat anti-Rabbit IgG- AF568	Thermo Fisher Scientific	Cat# A-11036; RRID: AB_10563566	1:600 for IF
Goat anti-Rabbit IgG- AF647	Thermo Fisher Scientific	Cat# A-21244; RRID: AB_2535812	1:600 for IF
Donkey anti-guinea pig IgG-DyLight405	Jackson ImmunoResearch	Cat# 706-475-148; RRID: AB_2340470	1:200 for IF
Goat anti-guinea pig IgG-AF488	Thermo Fisher Scientific	Cat# A-11073; RRID: AB_2534117	1:600 for IF
Goat anti-guinea pig IgG-AF568	Thermo Fisher Scientific	Cat# A-11075; RRID: AB_2534119	1:600 for IF
Goat anti-guinea pig IgG-AF647	Thermo Fisher Scientific	Cat# A-21450<br>RRID: AB_2735091	1:300 for IF
Donkey anti-guinea pig IgG-AF647	Jackson ImmunoResearch	Cat# 706-605-148<br>RRID: AB_2340476	1:300 for IF
Goat anti-mouse IgG-AF405	Thermo Fisher Scientific	Cat# A-31553; RRID: AB_221604	1:200 for IF
Goat anti-mouse IgG-AF488	Thermo Fisher Scientific	Cat# A-11029; RRID: AB_2534088	1:600 for IF
Goat anti-mouse IgG-AF568	Thermo Fisher Scientific	Cat# A-11031; RRID: AB_144696	1:600 for IF
Goat anti-chicken IgY-AF405	Abcam	Cat# ab175675<br>RRID: AB_2810980	1:200 for IF
Goat anti-chicken IgY-AF488	Thermo Fisher Scientific	Cat# A-11039; RRID: AB_2534096	1:600 for IF
Goat anti-chicken IgY-AF568	Thermo Fisher Scientific	Cat# A-11041; RRID: AB_2534098	1:600 for IF
Goat anti-rat IgG-AF488	Thermo Fisher Scientific	Cat# A-11006; RRID: AB_2534074	1:600 for IF
Chemicals, peptides, and recombinant proteins			
XL413 hydrochloride	Tocris	Cat# 5493<br>Cas# 1169562-71-3	
LY3143921 hydrate	SelleckChem	Cat# S9650<br>Cas# 1627696-53-0	

TAK-931 (Simurosertib)	MedChemExpress	Cat# HY-100888<br>Cas# 1330782-76-7	
Critical commercial assays			
CDC7/DBF4 kinase kinase assay	Promega	Cat# V5088	
Phostag Acrylamide	Fujifilm Wako Chemicals	Cat# AAL-107S1	
Experimental models: Organisms/strains			
Y2HGold Yeast strain	Clontech	Cat# 630498	
Mouse *Iho1*$^{C7\Delta/C7\Delta}$	This study		
Mouse/ *Ankrd31*$^{+/+}$ and *Ankrd31*$^{-/-}$	[2]		
Mouse/ *Iho1*$^{+/+}$ and *Iho1*$^{-/-}$	[4]		
Mouse/ *Hormad1*$^{+/+}$ and *Hormad 1*$^{-/-}$	[8]		
Mouse/ *Spo11*$^{+/+}$ and *Spo11*$^{-/-}$	[18]		
Mouse/ *Dmc1*$^{-/-}$	[19]		
Oligonucleotides			
Iho1AA_Fw2: CATGACCACCAGAAGCGTCA	Eurofins Genomics	N/A	
Iho1AA_Rv2: AATGTTTTCACCAAGGACATAC	Eurofins Genomics	N/A	
sgRNA: GGATTTTGATAGCAGCGATGATA	IDT	N/A	
Software and algorithms			
Jalview	[20]		
Cellprofiler 3.0	[21]		
ImageJ	[22, 23]		

Fiji Suite for ImageJ	[24]		
R: A language and environment for statistical computing.	[25]		
lmerTest Package	[26]		
Lme4 package	[27]		
Clustal Omega	[28, 29]		
Tubingen Bioinformatic Toolkit	[30, 31]		
Other			
salmon sperm	Thermo Fisher Scientific	Cat# AM9680	

Supplementary References:

1. Nore, A. *et al.* TOPOVIBL-REC114 interaction regulates meiotic DNA double-strand breaks. *Nature communications* **13**, 7048 (2022).
2. Papanikos, F. *et al.* Mouse ANKRD31 Regulates Spatiotemporal Patterning of Meiotic Recombination Initiation and Ensures Recombination between X and Y Sex Chromosomes. *Molecular cell* **74**, 1069-1085 e1011 (2019).
3. Boekhout, M. *et al.* REC114 Partner ANKRD31 Controls Number, Timing, and Location of Meiotic DNA Breaks. *Molecular cell* **74**, 1053-1068 e1058 (2019).
4. Stanzione, M. *et al.* Meiotic DNA break formation requires the unsynapsed chromosome axis-binding protein IHO1 (CCDC36) in mice. *Nature cell biology* **18**, 1208-1220 (2016).
5. Robert, T. *et al.* The TopoVIB-Like protein family is required for meiotic DNA double-strand break formation. *Science* **351**, 943-949 (2016).
6. Kumar, R., Bourbon, H.M. & de Massy, B. Functional conservation of Mei4 for meiotic DNA double-strand break formation from yeasts to mice. *Genes & development* **24**, 1266-1280 (2010).
7. Dereli, I. *et al.* Four-pronged negative feedback of DSB machinery in meiotic DNA-break control in mice. *Nucleic acids research* **49**, 2609-2628 (2021).
8. Daniel, K. *et al.* Meiotic homologue alignment and its quality surveillance are controlled by mouse HORMAD1. *Nature Cell Biology* **13**, 599-U232 (2011).
9. Wojtasz, L. *et al.* Mouse HORMAD1 and HORMAD2, Two Conserved Meiotic Chromosomal Proteins, Are Depleted from Synapsed Chromosome Axes with the Help of TRIP13 AAA-ATPase. *PLoS genetics* **5** (2009).
10. Tesse, S. *et al.* Asy2/Mer2: an evolutionarily conserved mediator of meiotic recombination, pairing, and global chromosome compaction. *Genes & development* **31**, 1880-1893 (2017).
11. Vrielynck, N. *et al.* Conservation and divergence of meiotic DNA double strand break forming mechanisms in Arabidopsis thaliana. *Nucleic acids research* **49**, 9821-9835 (2021).
12. Rousova, D. *et al.* Novel mechanistic insights into the role of Mer2 as the keystone of meiotic DNA break formation. *eLife* **10** (2021).

13. Kariyazono, R., Oda, A., Yamada, T. & Ohta, K. Conserved HORMA domain-containing protein Hop1 stabilizes interaction between proteins of meiotic DNA break hotspots and chromosome axis. *Nucleic acids research* **47**, 10166-10180 (2019).
14. Daccache, D. *et al.* Evolutionary conservation of the structure and function of meiotic Rec114-Mei4 and Mer2 complexes. *Genes & development* **37**, 535-553 (2023).
15. Heldrich, J. *et al.* Two pathways drive meiotic chromosome axis assembly in Saccharomyces cerevisiae. *Nucleic acids research* **50**, 4545-4556 (2022).
16. Finsterbusch, F. *et al.* Alignment of Homologous Chromosomes and Effective Repair of Programmed DNA Double-Strand Breaks during Mouse Meiosis Require the Minichromosome Maintenance Domain Containing 2 (MCMDC2) Protein. *PLoS genetics* **12**, e1006393 (2016).
17. Offenberg, H.H., Dietrich, A.J. & Heyting, C. Tissue distribution of two major components of synaptonemal complexes of the rat. *Chromosoma* **101**, 83-91 (1991).
18. Baudat, F., Manova, K., Yuen, J.P., Jasin, M. & Keeney, S. Chromosome synapsis defects and sexually dimorphic meiotic progression in mice lacking Spo11. *Molecular cell* **6**, 989-998 (2000).
19. Pittman, D.L. *et al.* Meiotic prophase arrest with failure of chromosome synapsis in mice deficient for *Dmc1*, a germline-specific RecA homolog. *Molecular cell* **1**, 697-705 (1998).
20. Waterhouse, A.M., Procter, J.B., Martin, D.M., Clamp, M. & Barton, G.J. Jalview Version 2--a multiple sequence alignment editor and analysis workbench. *Bioinformatics* **25**, 1189-1191 (2009).
21. McQuin, C. *et al.* CellProfiler 3.0: Next-generation image processing for biology. *PLoS biology* **16**, e2005970 (2018).
22. Rueden, C.T. *et al.* ImageJ2: ImageJ for the next generation of scientific image data. *BMC bioinformatics* **18**, 529 (2017).
23. Schneider, C.A., Rasband, W.S. & Eliceiri, K.W. NIH Image to ImageJ: 25 years of image analysis. *Nature methods* **9**, 671-675 (2012).
24. Schindelin, J. *et al.* Fiji: an open-source platform for biological-image analysis. *Nature methods* **9**, 676-682 (2012).
25. Team, R.C. (2022).
26. Kuznetsova, A., Brockhoff, P.B. & Christensen, R.H.B. lmerTest Package: Tests in Linear Mixed Effects Models. *Journal of Statistical Software* **82**, 1-26 (2017).
27. Bates D., M.M., Bolker B.M., Walker S.C. Fitting linear mixed-effects models using lme4. *Journal of Statistical Software* **67**, 1-48 (2015).
28. McWilliam, H. *et al.* Analysis Tool Web Services from the EMBL-EBI. *Nucleic acids research* **41**, W597-600 (2013).
29. Sievers, F. *et al.* Fast, scalable generation of high-quality protein multiple sequence alignments using Clustal Omega. *Mol Syst Biol* **7**, 539 (2011).
30. Gabler, F. *et al.* Protein Sequence Analysis Using the MPI Bioinformatics Toolkit. *Curr Protoc Bioinformatics* **72**, e108 (2020).
31. Zimmermann, L. *et al.* A Completely Reimplemented MPI Bioinformatics Toolkit with a New HHpred Server at its Core. *J Mol Biol* **430**, 2237-2243 (2018).
