# Supplementary material for: Seeding the meiotic DNA break machinery and initiating recombination on chromosome axes
Source: Nat Commun. 2024 Apr 5;15:2941. doi: 10.1038/s41467-024-47020-1 (PMC10997794; doi:10.1038/s41467-024-47020-1)
Supplement: Supplementary file 3 — Reporting Summary [file 41467_2024_47020_MOESM3_ESM.pdf]

nature portfolio

Corresponding author(s): Attila Tóth

Last updated by author(s): 2024/03/03

Reporting Summary

Nature Portfolio wishes to improve the reproducibility of the work that we publish. This form provides structure for consistency and transparency in reporting. For further information on Nature Portfolio policies, see our Editorial Policies and the Editorial Policy Checklist.

Statistics

For all statistical analyses, confirm that the following items are present in the figure legend, table legend, main text, or Methods section.

n/a	Confirmed	
☐	☒	The exact sample size (*n*) for each experimental group/condition, given as a discrete number and unit of measurement
☐	☒	A statement on whether measurements were taken from distinct samples or whether the same sample was measured repeatedly
☐	☒	The statistical test(s) used AND whether they are one- or two-sided<br>*Only common tests should be described solely by name; describe more complex techniques in the Methods section.*
☒	☐	A description of all covariates tested
☐	☒	A description of any assumptions or corrections, such as tests of normality and adjustment for multiple comparisons
☐	☒	A full description of the statistical parameters including central tendency (e.g. means) or other basic estimates (e.g. regression coefficient) AND variation (e.g. standard deviation) or associated estimates of uncertainty (e.g. confidence intervals)
☐	☒	For null hypothesis testing, the test statistic (e.g. *F*, *t*, *r*) with confidence intervals, effect sizes, degrees of freedom and *P* value noted<br>*Give P values as exact values whenever suitable.*
☒	☐	For Bayesian analysis, information on the choice of priors and Markov chain Monte Carlo settings
☒	☐	For hierarchical and complex designs, identification of the appropriate level for tests and full reporting of outcomes
☒	☐	Estimates of effect sizes (e.g. Cohen's *d*, Pearson's *r*), indicating how they were calculated

Our web collection on statistics for biologists contains articles on many of the points above.

Software and code

Policy information about availability of computer code

Data collection	Image data collection was performed using AxioVision version 4.8 or Zen 2.3 Pro software.
Data analysis	GraphPad Prism 9, R version 4.1.3, Geneious 5.6.3, Fiji-Imagej 1.54f, Adobe Photoshop CC19, Clustal Omega tool, Jalview version 2, Proteome Discoverer 2.5, Cell Profiler 3.0

For manuscripts utilizing custom algorithms or software that are central to the research but not yet described in published literature, software must be made available to editors and reviewers. We strongly encourage code deposition in a community repository (e.g. GitHub). See the Nature Portfolio guidelines for submitting code & software for further information.

Data

Policy information about availability of data

All manuscripts must include a data availability statement. This statement should provide the following information, where applicable:

- Accession codes, unique identifiers, or web links for publicly available datasets
- A description of any restrictions on data availability
- For clinical datasets or third party data, please ensure that the statement adheres to our policy

The authors declare that the data supporting the findings of this study are available within the paper and its Supplementary Information. The mass spectrometry proteomics data have been deposited to the ProteomeXchange Consortium via the PRIDE 113 partner repository with the dataset identifiers PXD042179

(hyperlink: http://www.ebi.ac.uk/pride/archive/projects/PXD042179) and PXD042221 (hyperlink: http://www.ebi.ac.uk/pride/archive/projects/PXD042221). All other data supporting the findings of this study are available from the corresponding author upon request. Source data are provided with this paper.

Research involving human participants, their data, or biological material

Policy information about studies with human participants or human data. See also policy information about sex, gender (identity/presentation), and sexual orientation and race, ethnicity and racism.

Reporting on sex and gender	Not relevant for this study.
Reporting on race, ethnicity, or other socially relevant groupings	Not relevant for this study.
Population characteristics	Not relevant for this study.
Recruitment	Not relevant for this study.
Ethics oversight	Not relevant for this study.

Note that full information on the approval of the study protocol must also be provided in the manuscript.

Field-specific reporting

Please select the one below that is the best fit for your research. If you are not sure, read the appropriate sections before making your selection.

☒ Life sciences ☐ Behavioural & social sciences ☐ Ecological, evolutionary & environmental sciences

For a reference copy of the document with all sections, see nature.com/documents/nr-reporting-summary-flat.pdf

Life sciences study design

All studies must disclose on these points even when the disclosure is negative.

Sample size	No formal sample-size calculations were performed. Nevertheless, sample sizes were chosen based on our past experiences and publications in the field to allow the detection of medium sized effects (equivalent to Cohen´s d 0.4-0.5) with confidence. Given that the examined mutant mice had very severe phenotypes in recombination, meiocyte apoptosis and fertility, the chosen sample sizes were appropriate and justified. For examples see the following papers: Wojtasz et. al. Meiotic DNA double-strand breaks and chromosome asynapsis in mice are monitored by distinct HORMAD2-independent and -dependent mechanisms. Genes Dev 26, 958-973 (2012), Stanzione, M. et al. Meiotic DNA break formation requires the unsynapsed chromosome axis-binding protein IHO1 (CCDC36) in mice. Nat Cell Biol 18, 1208–1220 (2016); Qiao, H. et al. Antagonistic roles of ubiquitin ligase HEI10 and SUMO ligase RNF212 regulate meiotic recombination. Nat Genet 46, 194–199 (2014); Zhang, J. et al. The BRCA2-MEILB2-BRME1 complex governs meiotic recombination and impairs the mitotic BRCA2-RAD51 function in cancer cells. Nat Commun 11, 2055 (2020); Holloway, J.K. et al. Mammalian CNTD1 is critical for meiotic crossover maturation and deselection of excess precrossover sites. J Cell Biol 205 (5), 633–641 (2014).
Data exclusions	No data were excluded from the analyses.
Replication	All phenotypes were observed in at least two animals of each genotype and, unless stated otherwise, quantifications represent analysis of at least two independent animals. All comparisons were made between datasets obtained from animals that were either littermates or matched by age.<br>All yeast-two hybrid assay results were reproduced in at least two independent repetitions of experiments.<br>Replications attempts were successful and produced consistent results both in experiments that involved animals and experiments that did not.
Randomization	Specific randomization methods are not relevant to the study. The study relies on comparison of wild type and various mutant mice that were generated by random segregation of alleles during sexual reproduction, which ensures random allocation of samples. Where control versus mutant mice were compared, samples were processed in parallel to eliminate batch effects.
Blinding	Blinding is not relevant to the study as, due to the drastic differences in phenotypes of wild type and mutant animals, even blinded investigator would be able to distinguish between the control and mutant samples. Yeast-two hybrid experiments are well controlled and have an unambiguous visual readout that is not influenced by the experimenter´s knowledge about sample identity. Images representing raw results are also presented in the figures of the manuscript, allowing direct evaluation by readers. Hence, blinding is not necessary.<br>All comparisons in yeast-two hybrid experiments were made between yeast transformed in the same experiment and grown on the same plate, so that the samples can be compared side-by-side without the need for blinding the investigator.

Reporting for specific materials, systems and methods

We require information from authors about some types of materials, experimental systems and methods used in many studies. Here, indicate whether each material, system or method listed is relevant to your study. If you are not sure if a list item applies to your research, read the appropriate section before selecting a response.

Materials & experimental systems

n/a	Involved in the study
☐	☒ Antibodies
☒	☐ Eukaryotic cell lines
☒	☐ Palaeontology and archaeology
☐	☒ Animals and other organisms
☒	☐ Clinical data
☒	☐ Dual use research of concern
☒	☐ Plants

Methods

n/a	Involved in the study
☒	☐ ChIP-seq
☒	☐ Flow cytometry
☒	☐ MRI-based neuroimaging

Antibodies

Antibodies used

All the antibodies used in the study are described in a Table of reagents and resources in the Supplementary information. The list of antibodies is as follows:Rabbit polyclonal anti-GFP Thermo Fisher Scientific Cat# A-11122
Rabbit polyclonal anti-SYCP3 Abcam Cat# ab15093
Mouse monoclonal anti- β-Actin Santa Cruz Cat# ab47778
Mouse monoclonal anti-GAPDH Santa Cruz Cat# sc-32233
Rabbit polyclonal anti-Histone H3 Abcam Cat# ab1791
Mouse monoclonal anti-α-TUBULIN Sigma-Aldrich Cat# T6199
Rabbit polyclonal anti-DMC1 (H-100) Santa Cruz Cat# sc-22768
Rabbit polyclonal anti-Rad51 (H-92) Santa Cruz Cat# sc-8349
Rat monoclonal anti-RPA32 (4E4) Cell signaling Cat# 2208
Rabbit polyclonal anti-cleaved PARP (Asp214) Cell signaling Cat# 9544
Mouse monoclonal anti-phospho-Histone H2A.X (Ser139) Millipore Cat# 05-636
Rabbit polyclonal anti-MVH Abcam Cat# ab13840
Mouse monoclonal anti-p63 Biocare Medical Cat# CM163A
Mouse monoclonal anti-SPO11(Spo11-180) MSKCC Antibody and Bioresource Core Facility N/A
Rabbit polyclonal anti-SYCP1 Abcam Cat# ab15090
Mouse monoclonal anti-MLH1 Cell signaling Cat# 3515S
Goat anti-rabbit IgG-HRP Jackson ImmunoResearch Cat# 111-035-003
Goat anti-guinea pig IgG-HRP Jackson ImmunoResearch Cat# 706-035-148
Goat anti-mouse IgG-HRP Jackson ImmunoResearch Cat# 115-035-003
Goat anti-Rabbit IgG-AF405 Thermo Fisher Scientific Cat# A-31556
Goat anti-Rabbit IgG-AF488 Thermo Fisher Scientific Cat# A-11034
Goat anti-Rabbit IgG- AF568 Thermo Fisher Scientific Cat# A-11036
Goat anti-Rabbit IgG- AF647 Thermo Fisher Scientific Cat# A-21244
Donkey anti-guinea pig IgG-DyLight405 Jackson ImmunoResearch Cat# 706-475-148
Goat anti-guinea pig IgG-AF488 Thermo Fisher Scientific Cat# A-11073
Goat anti-guinea pig IgG-AF568 Thermo Fisher Scientific Cat# A-11075
Goat anti-guinea pig IgG-AF647 Thermo Fisher Scientific Cat# A-21450
Donkey anti-guinea pig IgG-AF647 Jackson ImmunoResearch Cat# 706-605-148
Goat anti-mouse IgG-AF405 Thermo Fisher Scientific Cat# A-31553
Goat anti-mouse IgG-AF488 Thermo Fisher Scientific Cat# A-11029
Goat anti-mouse IgG-AF568 Thermo Fisher Scientific Cat# A-11031
Goat anti-chicken IgY-AF405 Abcam Cat# ab175675
Goat anti-chicken IgY-AF488 Thermo Fisher Scientific Cat# A-11039
Goat anti-chicken IgY-AF568 Thermo Fisher Scientific Cat# A-11041
Goat anti-rat IgG-AF488 Thermo Fisher Scientific Cat# A-11006

Validation

Earlier published antibodies have been already validated in the published papers. Additionally, commecially available and earlier published antibodies used for immunofluorescence were validated by us in immunostaining by comparing their staining patterns with the known localisation patterns of the respective proteins in meiocytes or on the gonads sections. This type of validation was applied to antibodies as follows: Ch anti-SYCP3 and anti-SYCP1 (A Toth), Mm anti-SYCP3 (R. Jessberger), Gp and Rb anti-IHO1 (A. Toth), Gp and Rb anti-HORMAD1 (A. Toth), Gp anti-MEI4 (A. Toth), Rb anti-REC114 (A. Toth), Gp and Rb anti-H1t (A. Toth), Mm anti-MLH1 (Cell Signaling, #3515), Mm anti-γH2AX (Millipore, #05-636), Rat anti-RPA32/RPA2 (Cell Signaling, #2208), Rb anti-DMC1 (Santa Cruz, sc-22768), Rb anti-RAD51 (Santa Cruz, sc-8349), Rb anti-DDX4/MVH (Abcam, ab13840), Mm anti-p63 (Biocare Medical, CM163A). Specificity of Rb anti-GFP antibody was validated by confirming positive and negative signal on spermatocytes transfected with plasmids harboring GFP tagged IHO1 or empty plasmid, respectively. Antibodies used in western blot were validated by us in immunoblot analysis of testis extracts by comparing the electrophoretic mobility of detected protein bands with the known/ published electrophoretic mobility of respective proteins: Rb anti-histone H3 (Abcam, ab18521, for validation by manufacturer for WB see https://www.abcam.com/en-de/search?facets.categoryType=Primary+Antibodies&sorting=relevance&keywords=ab18521), Mm anti-GAPDH (Santa Cruz, sc-32233, for validation by manufacturer for WB see https://www.scbt.com/p/gapdh-antibody-6c5?requestFrom=search), Mm anti-α-tubulin (Sigma, T6199, for validation by manufacturer for WB see https://www.sigmaaldrich.com/DE/en/product/sigma/t6199) and Mm anti- β-Actin (Santa Cruz, 47778, for validation by manufacturer for WB see https://www.scbt.com/p/beta-actin-antibody-c4?requestFrom=search). Additional citations relevant to antibody validations are as follows: Ch anti-SYCP3 Finsterbusch, F. et al. PLoS genetics 12, e1006393 (2016). Mm anti-SYCP3 Offenberg, H.H., Dietrich, A.J. & Heyting, C.

	Chromosoma 101, 83-91 (1991). Ch anti-SYCP1, Rb anti-MEI4, Gp and Rb anti-H1t Papanikos, F. et al .Molecular cell 74, 1069-1085 e1011 (2019). Gp and Rb anti-IHO1, Gp anti-MEI4 Stanzione, M. et al. Nature cell biology 18, 1208-1220 (2016). Gp and Rb anti-HORMAD1 Wojtasz, L. et al. PLoS genetics 5 (2009).

Animals and other research organisms

Policy information about studies involving animals; ARRIVE guidelines recommended for reporting animal research, and Sex and Gender in Research

Laboratory animals	Mouse strains used in the study: Iho1C7Δ/C7Δ, Ankrd31−/−, Iho1−/−, Dmc1 -/-, Hormad1 -/- and Spo11 -/-. Adult male and female C57BL/6JCrl mice were used for breeding Iho1C7Δ/C7Δs in a defined background. Gonads were collected from mice after euthanasia. Cytological experiments were carried out on samples collected from adult male, adult female mice and female foetuses (16 and 18 days post coitum), . Protein extracts were collected from testes of 13 days old male pups. For testis organ cultures, 8 dpp male mice were used. Typically, adult mice were used between 60-150 days of age except for ovary sections where 6 weeks old females were used.<br>The mice were kept in the barrier facility in individually ventilated cages at 22–24°C and 50–55% air humidity with 14-h light/10-h dark cycle. The feed was a rat–mouse standard diet in the form of pellets. The stocking density in the used cage type IIL was maximum five mice. Hygiene monitoring was carried out according to FELASA guidelines.
Wild animals	The study did not involve wild animals.
Reporting on sex	Both sexes were analyzed separately because male and female meiosis in mice take place at different developmental stages and are controlled by sex-specific mechanisms in addition to common pathways regulating mammalian meiosis. Male and female data were collected and reported separately.
Field-collected samples	The study did not involve samples collected from the field.
Ethics oversight	All mice were used and maintained in accordance with the German Animal Welfare legislation (“Tierschutzgesetz”). All procedures pertaining to animal experiments were approved by the Governmental IACUC (“Landesdirektion Sachsen”) and overseen by the animal ethics committee of the Technische Universität Dresden. The licence numbers concerned with the present experiments with mice are TVV 2014/17, TV A 8/2017, TV A 23/2017, and TV vG 3/2022.

Note that full information on the approval of the study protocol must also be provided in the manuscript.

Plants

Seed stocks	Not relevant for this study.
Novel plant genotypes	Not relevant for this study.
Authentication	Not relevant for this study.
